# Supplementary material for: Plasma Lipidomic Profiling Using Mass Spectrometry for Multiple Sclerosis Diagnosis and Disease Activity Stratification (LipidMS)
Source: Int J Mol Sci. 2024 Feb 20;25(5):2483. doi: 10.3390/ijms25052483 (PMC10932002; doi:10.3390/ijms25052483)
Supplement: Supplementary file 1 [file ijms-25-02483-s001.zip › LSI-Checklist.pdf]

Direct Infusion Workflow

Created by https://lipidomicstandards.org, version v1.0.0

Overall study design

Title of the study	Plasma Lipidomic Profiling by Mass Spectrometry for Multiple Sclerosis Diagnosis and Disease Activity Stratification (LipidMS)		
Document creation date	09/28/2023	Corresponding Email	dirk.fitzner@med.uni-goettigen.de
Principle investigator	PD Dr. med Dirk Fitzner	Is the workflow targeted or untargeted?	Untargeted
Institution	University Hospital Goettingen, Germany	Clinical	Yes

Lipid extraction

Extraction method	2-phase system	2-phase system	MTBE
pH adjustment	None	Were internal standards added prior extraction?	Yes

Analytical platform

MS type	Orbitrap	Mass accuracy in ppm at MS1	1
MS vendor	Thermo	Mass window for precursor ion isolation (in Da total isolation window)	1
Ion source	ESI	Mass resolution for detected ion at MS2	High resolution
Direct type	Chip	Resolution at m/z 200 at MS2	17500
MS Level	MS1, MS2	Mass accuracy in ppm at MS2	3
Mass resolution for detected ion at MS1	High resolution	Was/Were additional dimension/techniques used	No
Resolution at m/z 200 at MS1	280000		

Quality control

Blanks	Yes	Quality control	Yes
Type of Blanks	Extraction blank	Type of QC sample	Reference material

Method qualification and validation

Method validation	Yes	Precison	Yes
Lipid recovery	Yes	Accuracy	No
Dynamic quantification range	Yes	Guidelines followed	None
Limit of quantitation (LOQ)/Limit of detection (LOD)	Yes		

Reporting

Are reported raw data uploaded into repository?	Available on request	Additional comments	-
Raw data upload	No		

Sample Descriptions

Plasma samples / Human / Plasma

Provided information	-	Additives	None
Temperature handling original sample	4-8 °C	Were samples stored under inert gas?	No
Instant sample preparation	No	Additional preservation methods	No
Storage temperature	-80 °C	Biobank samples	Yes

Lipid Class Descriptions

1) LPC[M+CH3COO]- / Lipid identification

Lipid class	LPC	Check isomer overlap	Yes
MS Level for identification	MS1	Additional dimension/techniques	-
Identification level	Species level	Lipid Identification Software	LipotypeXplorer
Polarity mode	Negative	Data manipulation	Centroiding
Type of negative (precursor)ion	[M+CH3COO]-	Nomenclature for intact lipid molecule	Yes

1) LPC[M+CH3COO]- / For additional separation methods/analytical dimension

Quantitative	Yes	Limit of quantification	S/N ratio
MS Level for quantification	MS1	Normalization to reference	No
Type of quantification	Internal standard amount	Lipid Quantification Software	LipotypeXplorer
Response correction	No	Batch correction	Normalization by reference material/QC
Type I isotope correction	Yes		

2) Cer[M+CH3COO]- / Lipid identification

Lipid class	Cer	Check isomer overlap	Yes
MS Level for identification	MS1	Additional dimension/techniques	-
Identification level	Species level	Lipid Identification Software	LipotypeXplorer
Polarity mode	Negative	Data manipulation	Centroiding
Type of negative (precursor)ion	[M+CH3COO]-	Nomenclature for intact lipid molecule	Yes

2) Cer[M+CH3COO]- / For additional separation methods/analytical dimension

Quantitative	Yes	Limit of quantification	S/N ratio
MS Level for quantification	MS1	Normalization to reference	No
Type of quantification	Internal standard amount	Lipid Quantification Software	LipotypeXplorer
Response correction	No	Batch correction	Normalization by reference material/QC
Type I isotope correction	Yes		

3) SM[M+CH3COO]- / Lipid identification

Lipid class	SM	Check isomer overlap	Yes
MS Level for identification	MS1	Additional dimension/techniques	-
Identification level	Species level	Lipid Identification Software	LipotypeXplorer
Polarity mode	Negative	Data manipulation	Centroiding
Type of negative (precursor)ion	[M+CH3COO]-	Nomenclature for intact lipid molecule	Yes

3) SM[M+CH3COO]- / For additional separation methods/analytical dimension

Quantitative	Yes	Limit of quantification	S/N ratio
MS Level for quantification	MS1	Normalization to reference	No
Type of quantification	Internal standard amount	Lipid Quantification Software	LipotypeXplorer
Response correction	No	Batch correction	Normalization by reference material/QC
Type I isotope correction	Yes		

4) HexCer[M+CH3COO]- / Lipid identification

Lipid class	HexCer	Check isomer overlap	Yes
MS Level for identification	MS1	Additional dimension/techniques	-
Identification level	Species level	Lipid Identification Software	LipotypeXplorer
Polarity mode	Negative	Data manipulation	Centroiding
Type of negative (precursor)ion	[M+CH3COO]-	Nomenclature for intact lipid molecule	No

4) HexCer[M+CH3COO]- / For additional separation methods/analytical dimension

Quantitative	Yes	Limit of quantification	S/N ratio
MS Level for quantification	MS1	Normalization to reference	No
Type of quantification	Internal standard amount	Lipid Quantification Software	LipotypeXplorer
Response correction	No	Batch correction	Normalization by reference material/QC
Type I isotope correction	Yes		

5) LPC O-a[M+CH3COO]- / Lipid identification

Lipid class	LPC O-a	Check isomer overlap	Yes
MS Level for identification	MS1	Additional dimension/techniques	-
Identification level	Species level	Lipid Identification Software	LipotypeXplorer
Polarity mode	Negative	Data manipulation	Centroiding
Type of negative (precursor)ion	[M+CH3COO]-	Nomenclature for intact lipid molecule	No

5) LPC O-a[M+CH3COO]- / For additional separation methods/analytical dimension

Quantitative	Yes	Limit of quantification	S/N ratio
MS Level for quantification	MS1	Normalization to reference	No
Type of quantification	Internal standard amount	Lipid Quantification Software	LipotypeXplorer
Response correction	No	Batch correction	Normalization by reference material/QC
Type I isotope correction	Yes		

6) LPC O-p[M+CH3COO]- / Lipid identification

Lipid class	LPC O-p	Check isomer overlap	Yes
MS Level for identification	MS1	Additional dimension/techniques	-
Identification level	Species level	Lipid Identification Software	LipotypeXplorer
Polarity mode	Negative	Data manipulation	Centroiding
Type of negative (precursor)ion	[M+CH3COO]-	Nomenclature for intact lipid molecule	No

6) LPC O-p[M+CH3COO]- / For additional separation methods/analytical dimension

Quantitative	Yes	Limit of quantification	S/N ratio
MS Level for quantification	MS1	Normalization to reference	No
Type of quantification	Internal standard amount	Lipid Quantification Software	LipotypeXplorer
Response correction	No	Batch correction	Normalization by reference material/QC
Type I isotope correction	Yes		

7) LPE O-a[M-H]- / Lipid identification

Lipid class	LPE O-a	Check isomer overlap	Yes
MS Level for identification	MS1	Additional dimension/techniques	-
Identification level	Species level	Lipid Identification Software	LipotypeXplorer
Polarity mode	Negative	Data manipulation	Centroiding
Type of negative (precursor)ion	[M-H]-	Nomenclature for intact lipid molecule	No

7) LPE O-a[M-H]- / For additional separation methods/analytical dimension

Quantitative	Yes	Limit of quantification	S/N ratio
MS Level for quantification	MS1	Normalization to reference	No
Type of quantification	Internal standard amount	Lipid Quantification Software	LipotypeXplorer
Response correction	No	Batch correction	Normalization by reference material/QC
Type I isotope correction	Yes		

8) LPE O-p[M-H]- / Lipid identification

Lipid class	LPE O-p	Check isomer overlap	Yes
MS Level for identification	MS1	Additional dimension/techniques	-
Identification level	Species level	Lipid Identification Software	LipotypeXplorer
Polarity mode	Negative	Data manipulation	Centroiding
Type of negative (precursor)ion	[M-H]-	Nomenclature for intact lipid molecule	Yes

8) LPE O-p[M-H]- / For additional separation methods/analytical dimension

Quantitative	Yes	Limit of quantification	S/N ratio
MS Level for quantification	MS1	Normalization to reference	No
Type of quantification	Internal standard amount	Lipid Quantification Software	LipotypeXplorer
Response correction	No	Batch correction	Normalization by reference material/QC
Type I isotope correction	Yes		

9) LPE[M-H]- / Lipid identification

Lipid class	LPE	Check isomer overlap	Yes
MS Level for identification	MS1	Additional dimension/techniques	-
Identification level	Species level	Lipid Identification Software	LipotypeXplorer
Polarity mode	Negative	Data manipulation	Centroiding
Type of negative (precursor)ion	[M-H]-	Nomenclature for intact lipid molecule	No

9) LPE[M-H]- / For additional separation methods/analytical dimension

Quantitative	Yes	Limit of quantification	S/N ratio
MS Level for quantification	MS1	Normalization to reference	No
Type of quantification	Internal standard amount	Lipid Quantification Software	LipotypeXplorer
Response correction	No	Batch correction	Normalization by reference material/QC
Type I isotope correction	Yes		

10) ST 27:1;1[M+NH4]+ / Lipid identification

Lipid class	ST 27:1;1	Check isomer overlap	Yes
MS Level for identification	MS1	Additional dimension/techniques	-
Identification level	Species level	Lipid Identification Software	LipotypeXplorer
Polarity mode	Positive	Data manipulation	Centroiding
Type of positive (precursor)ion	[M+NH4]+	Nomenclature for intact lipid molecule	Yes

10) ST 27:1;1[M+NH4]+ / For additional separation methods/analytical dimension

Quantitative	Yes	Limit of quantification	S/N ratio
MS Level for quantification	MS1	Normalization to reference	No
Type of quantification	Internal standard amount	Lipid Quantification Software	LipotypeXplorer
Response correction	No	Batch correction	Normalization by reference material/QC
Type I isotope correction	Yes		

11) PC[M+CH3COO]- / Lipid identification

Lipid class	PC	Check isomer overlap	Yes
MS Level for identification	MS2	Additional dimension/techniques	-
Identification level	Molecular species level	Lipid Identification Software	LipotypeXplorer
Polarity mode	Negative	Data manipulation	Centroiding
Type of negative (precursor)ion	[M+CH3COO]-	Nomenclature for intact lipid molecule	Yes

11) PC[M+CH3COO]- / For additional separation methods/analytical dimension

Quantitative	Yes	Limit of quantification	S/N ratio
MS Level for quantification	MS2	Normalization to reference	No
Type of quantification	Internal standard amount	Lipid Quantification Software	LipotypeXplorer
Response correction	No	Batch correction	Normalization by reference material/QC
Type I isotope correction	Yes		

12) PC O-a[M+CH3COO]- / Lipid identification

Lipid class	PC O-a	Check isomer overlap	Yes
MS Level for identification	MS2	Additional dimension/techniques	-
Identification level	Molecular species level	Lipid Identification Software	LipotypeXplorer
Polarity mode	Negative	Data manipulation	Centroiding
Type of negative (precursor)ion	[M+CH3COO]-	Nomenclature for intact lipid molecule	Yes

12) PC O-a[M+CH3COO]- / For additional separation methods/analytical dimension

Quantitative	Yes	Limit of quantification	S/N ratio
MS Level for quantification	MS2	Normalization to reference	No
Type of quantification	Internal standard amount	Lipid Quantification Software	LipotypeXplorer
Response correction	No	Batch correction	Normalization by reference material/QC
Type I isotope correction	Yes		

13) PC O-p[M+CH3COO]- / Lipid identification

Lipid class	PC O-p	Check isomer overlap	Yes
MS Level for identification	MS2	Additional dimension/techniques	-
Identification level	Molecular species level	Lipid Identification Software	LipotypeXplorer
Polarity mode	Negative	Data manipulation	Centroiding
Type of negative (precursor)ion	[M+CH3COO]-	Nomenclature for intact lipid molecule	Yes

13) PC O-p[M+CH3COO]- / For additional separation methods/analytical dimension

Quantitative	Yes	Limit of quantification	S/N ratio
MS Level for quantification	MS2	Normalization to reference	No
Type of quantification	Internal standard amount	Lipid Quantification Software	LipotypeXplorer
Response correction	No	Batch correction	Normalization by reference material/QC
Type I isotope correction	Yes		

14) PE[M-H]- / Lipid identification

Lipid class	PE	Check isomer overlap	Yes
MS Level for identification	MS2	Additional dimension/techniques	-
Identification level	Molecular species level	Lipid Identification Software	LipotypeXplorer
Polarity mode	Negative	Data manipulation	Centroiding
Type of negative (precursor)ion	[M-H]-	Nomenclature for intact lipid molecule	Yes

14) PE[M-H]- / For additional separation methods/analytical dimension

Quantitative	Yes	Limit of quantification	S/N ratio
MS Level for quantification	MS2	Normalization to reference	No
Type of quantification	Internal standard amount	Lipid Quantification Software	LipotypeXplorer
Response correction	No	Batch correction	Normalization by reference material/QC
Type I isotope correction	Yes		

15) PE O-a[M-H]- / Lipid identification

Lipid class	PE O-a	Check isomer overlap	Yes
MS Level for identification	MS2	Additional dimension/techniques	-
Identification level	Molecular species level	Lipid Identification Software	LipotypeXplorer
Polarity mode	Negative	Data manipulation	Centroiding
Type of negative (precursor)ion	[M-H]-	Nomenclature for intact lipid molecule	Yes

15) PE O-a[M-H]- / For additional separation methods/analytical dimension

Quantitative	Yes	Limit of quantification	S/N ratio
MS Level for quantification	MS2	Normalization to reference	No
Type of quantification	Internal standard amount	Lipid Quantification Software	LipotypeXplorer
Response correction	No	Batch correction	Normalization by reference material/QC
Type I isotope correction	Yes		

16) PE O-p[M-H]- / Lipid identification

Lipid class	PE O-p	Check isomer overlap	Yes
MS Level for identification	MS2	Additional dimension/techniques	-
Identification level	Molecular species level	Lipid Identification Software	LipotypeXplorer
Polarity mode	Negative	Data manipulation	Centroiding
Type of negative (precursor)ion	[M-H]-	Nomenclature for intact lipid molecule	Yes

16) PE O-p[M-H]- / For additional separation methods/analytical dimension

Quantitative	Yes	Limit of quantification	S/N ratio
MS Level for quantification	MS2	Normalization to reference	No
Type of quantification	Internal standard amount	Lipid Quantification Software	LipotypeXplorer
Response correction	No	Batch correction	Normalization by reference material/QC
Type I isotope correction	Yes		

17) PI[M-H]- / Lipid identification

Lipid class	PI	Check isomer overlap	Yes
MS Level for identification	MS2	Additional dimension/techniques	-
Identification level	Molecular species level	Lipid Identification Software	LipotypeXplorer
Polarity mode	Negative	Data manipulation	Centroiding
Type of negative (precursor)ion	[M-H]-	Nomenclature for intact lipid molecule	Yes

17) PI[M-H]- / For additional separation methods/analytical dimension

Quantitative	Yes	Limit of quantification	S/N ratio
MS Level for quantification	MS2	Normalization to reference	No
Type of quantification	Internal standard amount	Lipid Quantification Software	LipotypeXplorer
Response correction	No	Batch correction	Normalization by reference material/QC
Type I isotope correction	Yes		

18) SE 27:1[M+NH4]+ / Lipid identification

Lipid class	SE 27:1	Check isomer overlap	Yes
MS Level for identification	MS2	Additional dimension/techniques	-
Identification level	Molecular species level	Lipid Identification Software	LipotypeXplorer
Polarity mode	Positive	Data manipulation	Centroiding
Type of positive (precursor)ion	[M+NH4]+	Nomenclature for intact lipid molecule	Yes

18) SE 27:1[M+NH4]+ / For additional separation methods/analytical dimension

Quantitative	Yes	Limit of quantification	S/N ratio
MS Level for quantification	MS2	Normalization to reference	No
Type of quantification	Internal standard amount	Lipid Quantification Software	LipotypeXplorer
Response correction	No	Batch correction	Normalization by reference material/QC
Type I isotope correction	Yes		

19) DG[M+NH4]+ / Lipid identification

Lipid class	DG	Check isomer overlap	Yes
MS Level for identification	MS2	Additional dimension/techniques	-
Identification level	Molecular species level	Lipid Identification Software	LipotypeXplorer
Polarity mode	Positive	Data manipulation	Centroiding
Type of positive (precursor)ion	[M+NH4]+	Nomenclature for intact lipid molecule	Yes

19) DG[M+NH4]+ / For additional separation methods/analytical dimension

Quantitative	Yes	Limit of quantification	S/N ratio
MS Level for quantification	MS2	Normalization to reference	No
Type of quantification	Internal standard amount	Lipid Quantification Software	LipotypeXplorer
Response correction	No	Batch correction	Normalization by reference material/QC
Type I isotope correction	Yes		

20) TG[M+NH4]+ / Lipid identification

Lipid class	TG	Check isomer overlap	Yes
MS Level for identification	MS2	Additional dimension/techniques	-
Identification level	Molecular species level	Lipid Identification Software	LipotypeXplorer
Polarity mode	Positive	Data manipulation	Centroiding
Type of positive (precursor)ion	[M+NH4]+	Nomenclature for intact lipid molecule	Yes

20) TG[M+NH4]+ / For additional separation methods/analytical dimension

Quantitative	Yes	Limit of quantification	S/N ratio
MS Level for quantification	MS2	Normalization to reference	No
Type of quantification	Internal standard amount	Lipid Quantification Software	LipotypeXplorer
Response correction	No	Batch correction	Normalization by reference material/QC
Type I isotope correction	Yes		
