# Supplementary material for: Plasma Lipidomic Profiling Using Mass Spectrometry for Multiple Sclerosis Diagnosis and Disease Activity Stratification (LipidMS)
Source: Int J Mol Sci. 2024 Feb 20;25(5):2483. doi: 10.3390/ijms25052483 (PMC10932002; doi:10.3390/ijms25052483)
Supplement: Supplementary file 1 [file ijms-25-02483-s001.zip › Supplementary Materials.pdf]

Supplement:
Plasma Lipidomic Profiling using Mass Spectrometry for Multiple Sclerosis Diagnosis and Disease Activity Stratification (LipidMS)

Seyed Siyawasch Justus Lattau[1], Lisa-Marie Borsch[1], Kristina auf dem Brinke[1], Christian Klose[2], Liza Vinhoven[3], Manuel Nietert[3], Dirk Fitzner[1*]

[1] Department of Neurology, University Medical Center Göttingen, 37075 Göttingen, Germany
[2] Lipotype GmbH, 01307 Dresden, Germany
[3] Department of Medical Bioinformatics, University Medical Center Göttingen, 37075 Göttingen, Germany
[*] Correspondence:
PD Dr. med. D. Fitzner
Department of Neurology. University of Göttingen. Germany
Phone: +49 551 3967087
Email: d.fitzner@med.uni-goettingen.de

Table of Contents

SUPPLEMENT - METHODS 2
SUPPLEMENT - TABLES 3
SUPPLEMENT - FIGURES 22
REFERENCES: **Error! Bookmark not defined.**

Supplement- Table

SUPPLEMENT TABLE 1 AGE DISTRIBUTION ANALYSIS 3
SUPPLEMENT TABLE 2 BMI DISTRIBUTION ANALYSIS 4
SUPPLEMENT TABLE 3 STATISTICAL COMPARISONS OF LIPID CLASS AMOUNT 5
SUPPLEMENT TABLE 4 CHAIN-LENGTH ANALYSIS PART 1 6
SUPPLEMENT TABLE 5 CHAIN-LENGTH ANALYSIS PART 2 16
SUPPLEMENT TABLE 6 DISEASE MODIFYING THERAPY 20

Supplement- Figure

SUPPLEMENT FIGURE 1 DISTRIBUTION OF AGE AND BMI 22
SUPPLEMENT FIGURE 2 DATA IMPUTATION 23
SUPPLEMENT FIGURE 3 DISTRIBUTION OF LIPID SUBSPECIES 24
SUPPLEMENT FIGURE 4 LIPID-SPACE-CHART 25
SUPPLEMENT FIGURE 5 HEATMAP WITH HIERARCHICAL CLUSTERING 26
SUPPLEMENT FIGURE 6 SCATTER PLOTS OF LIPIDS WITH CORRELATION TO AGE AND BMI 27
SUPPLEMENT FIGURE 7 NONMS VS. MS COMPARISON 28
SUPPLEMENT FIGURE 8 OPLS-DA METRICS 29
SUPPLEMENT FIGURE 9 RECEIVER OPERATING CHARACTERISTIC CURVE (ROC-CURVE) OF THE RANDOM FOREST 30
SUPPLEMENT FIGURE 10 PERCENTAGE OF SIGNIFICANT ALTERED LIPIDS BY CLASS 31
SUPPLEMENT FIGURE 11 CHAIN-LENGTH ANALYSIS PLOT 32

Supplement- Methods

The data were analyzed using KNIME (v 4.5.2) and R (v 4.3.1). To address the issue of missing data, lipids that were absent in more than 90% of the measurements were removed from the analysis. Under the assumption that the missing data were predominantly due to left missingness, we performed imputation by replacing the missing values with zeros (Supplement Figure S2). The normalization of the measured lipids by logarithmic transformation, scaling (divides each lipid measurement by its standard deviation) and centering (subtracts the mean value from each lipid measurement) was required due to compositional nature of lipidomic data and for the application of statistical tests that assume a normal distribution (Supplement Figure S3). Since no normal distribution could be assumed for the calculation of the quantity differences of the chain lengths, a Kruskal-Wallis with Dunn post hoc test and Holm correction was performed (Supplement Table S4 and S5).

Lipids with a strong correlation with age or BMI were identified by comparing the healthy and OND cohorts filtering for Kendall's Tau Correlation ($r \leq |0.4|$) (Supplement Figure S1). These lipids were then marked separately in the further analysis. Principal Component Analysis (PCA) was generated by stats::prcomp. Prior to generating the clustered heatmap, the data underwent Min-Max normalization. Hierarchical clustering was then performed using the Manhattan distance metric and average linkage method (Supplement Figure 5). In addition to two group comparison using Welch's t-test, supervised machine learning was conducted using oPLS-DA. The oPLS-DA was generated by using ropls::opls(). Top 30 lipid species with most effect on oPLS-DA classification were determined using ropls::getVipVn(oplsda_model, orthoL = FALSE). The dataset was stratified to maintain the original distribution of the tested dataset and then split into a training part (60% of the data) and testing part (40% of the data). In these models one predictive component and one or two orthogonal components were calculated. The model fit was verified by evaluation of R2, which describes the variation of lipid species explained by the model and the cumulative R2X value (R2X(cum)) representing the explained variation in the matrix of predictor variables. R2Y(Cum), which represents the explained variation in the response matrix modeled by the predictor matrix was also calculated. The prediction ability of the model is described by Q2 and is calculated using 5-fold cross-validation (Q2(cum)). These values are shown in Supplement Figure 8. Additionally, a confusion matrix was calculated by using the testing dataset.

To assess the underlying diseases specific lipidomic changes between MS and non-MS samples, we used the Random Forest (RF) algorithm caret::train(cohort ~ ., data = train_data, method = "rf",). To reduce multicollinearity, we applied a Kendall correlation filter function from the caret package. The dataset was stratified to maintain the distribution of MS and non-MS participants and subsequently divided with 60% allocated to training and 40% to testing. In the first step, we utilized 5-fold cross-validation to tune the hyperparameters. We generated an ensemble of 1000 decision trees within the RF model. To ensure the robustness of the model's performance, we employed both hold-out validation on the testing dataset and 5-fold cross-validation (CV). The performance of the model was assessed using Receiver Operating Characteristic (ROC) analysis and confusion matrix. Furthermore, we calculated the lipids with the highest importance using the scaled mean decrease in accuracy method from caret::varImp(rf_model_cv_roc, type = 1 , scale = FALSE). This highlights the lipids that contribute the most to the model's ability to accurately distinguish between MS and non-MS samples by using the `Mean Decrease in Accuracy´.

Supplement- Tables

Supplement Table S1 Age Distribution Analysis

Comparison	W	p.value	d	n1	n2
healthy vs RRMS	214	0.00046	-1.145	30	30
healthy vs CPMS	11.5	<0.0001	-3.439	30	30
healthy vs OND	0	<0.0001	-3.91	30	30
RRMS vs CPMS	30	<0.0001	-2.911	30	30
RRMS vs OND	15	<0.0001	-3.203	30	30
CPMS vs OND	526.5	0.26069	0.245	30	30
inactive vs active	468.5	0.00351	0.912	47	13

The table presents the statistical evaluation of age distribution between the cohorts using the Mann-Whitney U test (MWU-Test).

W = sum of the ranks form MWU-Test; p.value = p. value from MWU-Test. p.values < 0.05 are considered statistically significant; d = Cohen's d; n1 and n2 = number of participants in each group.

Supplement Table S2 BMI Distribution Analysis

Comparison	W	p.value	d	n1	n2
healthy vs RRMS	361	0.19071	-0.503	30	30
healthy vs CPMS	319.5	0.0546	-0.51	30	30
healthy vs OND	202.5	0.00026	-1.089	30	30
RRMS vs CPMS	442	0.91237	0.128	30	30
RRMS vs OND	309	0.03778	-0.337	30	30
CPMS vs OND	310	0.03853	-0.563	30	30
inactive vs active	322	0.77668	0.062	47	13

The table presents the statistical evaluation of BMI distribution between the cohorts using the Mann-Whitney U test (MWU-Test).

W = sum of the ranks form MWU-Test; p.value = p. value from MWU-Test. p.values < 0.05 are considered statistically significant; d = Cohen's d; n1 and n2 = number of participants in each group.

Supplement Table S3 Statistical Comparisons of Lipid Class Amount

group1	group2	estimate	conf.low	conf.high	p.adj	p.adj.signif	Class
healthy	OND	779.0416281	88.18046887	1469.902787	0.0204	*	CE
CPMS	healthy	-12.86495357	-24.9151372	-0.814769935	0.0315	*	DAG
healthy	RRMS	14.47778435	2.427600718	26.52796799	0.0116	*	DAG
CPMS	healthy	10.21213734	1.158595172	19.26567952	0.0204	*	PI
healthy	RRMS	-9.945806245	-18.99934842	-0.892264072	0.0253	*	PI
OND	RRMS	-43.82209314	-83.33658168	-4.30760461	0.0234	*	SM
OND	RRMS	-1.607402615	-2.880984957	-0.333820274	0.00717	**	Cer
CPMS	OND	24.42774191	6.193549875	42.66193395	0.00374	**	PE
OND	RRMS	-23.00856428	-41.24275632	-4.774372244	0.00719	**	PE
CPMS	healthy	30.03512438	11.80093234	48.26931642	0.000213	***	PE
healthy	RRMS	-28.61594675	-46.85013879	-10.38175471	0.000458	***	PE
CPMS	healthy	-66.39716682	-105.9116554	-26.88267829	0.000152	***	SM
healthy	OND	2.33610184	1.062519498	3.609684181	0.0000303	****	Cer
CPMS	OND	21.86181073	10.90480712	32.81881433	0.00000512	****	PE O-
healthy	OND	25.47696267	14.51995906	36.43396627	0.000000103	****	PE O-
OND	RRMS	-25.96863772	-36.92564132	-15.01163411	0.0000000593	****	PE O-
healthy	OND	71.39498487	31.88049634	110.9094734	0.0000407	****	SM

*This table displays the lipid classes with significant alterations. as determined by ANOVA and Tukey correction for multiple comparisons; estimate represents the difference between the means of group1 and group2; conf.low and conf.high define the 95% confidence interval; p.adj is the p-value of the ANOVA after correction by TUKEY post hoc test; p.adj.signif indicates the level of significance; * indicates p < 0.05. ** indicates p < 0.01. *** indicates p < 0.001 **** indicates p < 0.0001.*

Cholesteryl ester (CE). Ceramide (Cer). Diacylglycerol (DAG). Phosphatidylethanolamine (PE). Ether-linked Phosphatidylethanolamine (PE O-) Phosphatidylinositol (PI) and Sphingomyelin (SM).

Chain-Length Analysis Chain 1

Supplement Table S4 Chain-Length Analysis Part 1

Comparison	Z	P.unadj	P.adj	Class	Chain
CPMS - healthy	-0.1712677	0.8640133	0.8640133	CE	14
CPMS - OND	-1.1276263	0.25947776	1	CE	14
healthy - OND	-0.9563587	0.33889106	1	CE	14
CPMS - RRMS	0.59190106	0.55391684	1	CE	14
healthy - RRMS	0.76316873	0.44536278	1	CE	14
OND - RRMS	1.71952739	0.08551839	0.51311031	CE	14
CPMS - healthy	-0.159588	0.87320566	1	CE	15
CPMS - OND	-0.3043305	0.76087608	1	CE	15
healthy - OND	-0.1447426	0.88491412	0.88491412	CE	15
CPMS - RRMS	2.14144785	0.03223794	0.12895176	CE	15
healthy - RRMS	2.30103582	0.02138961	0.10694803	CE	15
OND - RRMS	2.44577839	0.01445398	0.08672389	CE	15
CPMS - healthy	0.62851226	0.52966842	1	CE	16
CPMS - OND	-1.7908655	0.07331488	0.36657442	CE	16
healthy - OND	-2.4193777	0.01554709	0.09328252	CE	16
CPMS - RRMS	-0.2103807	0.83337058	0.83337058	CE	16
healthy - RRMS	-0.8388929	0.40152939	1	CE	16
OND - RRMS	1.58048481	0.11399588	0.45598353	CE	16
CPMS - healthy	0.41287207	0.67970034	0.67970034	CE	17
CPMS - OND	-1.0256058	0.30507744	0.61015488	CE	17
healthy - OND	-1.4384779	0.15029852	0.45089555	CE	17
CPMS - RRMS	2.18006973	0.02925229	0.14626147	CE	17
healthy - RRMS	1.76719766	0.07719513	0.30878053	CE	17
OND - RRMS	3.20567551	0.00134746	0.00808475	CE	17
CPMS - healthy	-0.1372821	0.89080783	1	CE	18
CPMS - OND	-0.6477853	0.51712383	1	CE	18
healthy - OND	-0.5105032	0.60969897	1	CE	18
CPMS - RRMS	0.09446869	0.92473685	0.92473685	CE	18
healthy - RRMS	0.23175077	0.81673159	1	CE	18
OND - RRMS	0.74225398	0.45793347	1	CE	18
CPMS - healthy	-0.5411675	0.58839212	1	CE	19
CPMS - OND	1.48164312	0.13843528	0.55374112	CE	19
healthy - OND	2.02281064	0.04309267	0.25855605	CE	19
CPMS - RRMS	-0.0277714	0.97784446	0.97784446	CE	19
healthy - RRMS	0.5133961	0.60767428	1	CE	19
OND - RRMS	-1.5094145	0.13119288	0.65596439	CE	19
CPMS - healthy	0.94867669	0.34278507	1	CE	20
CPMS - OND	-0.1856865	0.85269059	0.85269059	CE	20
healthy - OND	-1.1343632	0.25664222	1	CE	20
CPMS - RRMS	0.48329691	0.62888494	1	CE	20
healthy - RRMS	-0.4653798	0.64165951	1	CE	20
OND - RRMS	0.66898344	0.50350604	1	CE	20
CPMS - healthy	1.80027582	0.0718171	0.35908549	CE	22

CPMS - OND	-0.6962752	0.48625651	0.48625651	CE	22
healthy - OND	-2.496551	0.01254076	0.07524458	CE	22
CPMS - RRMS	0.9978259	0.31836379	0.95509137	CE	22
healthy - RRMS	-0.8024499	0.42229274	0.84458549	CE	22
OND - RRMS	1.69410108	0.09024608	0.36098431	CE	22
CPMS - healthy	-2.1270786	0.03341355	0.10024065	CE	23
CPMS - OND	-2.4591876	0.01392518	0.05570074	CE	23
healthy - OND	-0.3321089	0.73980701	0.73980701	CE	23
CPMS - RRMS	0.83817969	0.40192979	0.80385959	CE	23
healthy - RRMS	2.96525833	0.00302429	0.01512144	CE	23
OND - RRMS	3.29736726	0.00097596	0.00585575	CE	23
CPMS - healthy	-0.6097351	0.54203728	0.54203728	CE	24
CPMS - OND	-4.0088884	6.1005E-05	0.00030503	CE	24
healthy - OND	-3.3991533	0.00067595	0.00270379	CE	24
CPMS - RRMS	1.73798516	0.08221343	0.16442686	CE	24
healthy - RRMS	2.34772029	0.0188887	0.0566661	CE	24
OND - RRMS	5.74687359	9.0909E-09	5.4545E-08	CE	24
CPMS - healthy	1.18446434	0.2362293	0.70868791	Cer	40
CPMS - OND	-1.5624849	0.11817381	0.47269523	Cer	40
healthy - OND	-2.7469492	0.00601524	0.03609147	Cer	40
CPMS - RRMS	0.21673177	0.82841739	0.82841739	Cer	40
healthy - RRMS	-0.9677326	0.33317795	0.6663559	Cer	40
OND - RRMS	1.77921665	0.07520425	0.37602125	Cer	40
CPMS - healthy	0.9955718	0.31945824	1	Cer	42
CPMS - OND	-0.7224884	0.46999426	1	Cer	42
healthy - OND	-1.7180602	0.08578564	0.51471382	Cer	42
CPMS - RRMS	0.47234112	0.63668332	0.63668332	Cer	42
healthy - RRMS	-0.5232307	0.60081373	1	Cer	42
OND - RRMS	1.19482951	0.23215365	1	Cer	42
CPMS - healthy	0.18520847	0.85306553	1	Cer	44
CPMS - OND	0.45704671	0.64763747	1	Cer	44
healthy - OND	0.27183824	0.7857464	1	Cer	44
CPMS - RRMS	0.37340417	0.70884765	1	Cer	44
healthy - RRMS	0.1881957	0.85072324	1	Cer	44
OND - RRMS	-0.0836425	0.93334065	0.93334065	Cer	44
CPMS - healthy	-0.4738609	0.6355991	1	DAG	14
CPMS - OND	-1.8834976	0.05963297	0.29816486	DAG	14
healthy - OND	-1.4096366	0.158647	0.634588	DAG	14
CPMS - RRMS	0.44598674	0.65560683	0.65560683	DAG	14
healthy - RRMS	0.91984764	0.35765238	1	DAG	14
OND - RRMS	2.32948429	0.01983342	0.11900055	DAG	14
CPMS - healthy	6.17138671	6.7694E-10	4.0616E-09	DAG	16
CPMS - OND	4.46774493	7.9048E-06	3.1619E-05	DAG	16
healthy - OND	-1.7036418	0.08844803	0.17689606	DAG	16
CPMS - RRMS	0.64467434	0.51913825	0.51913825	DAG	16
healthy - RRMS	-5.5267124	3.2629E-08	1.6314E-07	DAG	16
OND - RRMS	-3.8230706	0.0001318	0.0003954	DAG	16

CPMS - healthy	3.61960168	0.00029506	0.00088517	DAG	18
CPMS - OND	3.80535925	0.0001416	0.00070799	DAG	18
healthy - OND	0.18575757	0.85263488	1	DAG	18
CPMS - RRMS	-0.1493987	0.88123903	0.88123903	DAG	18
healthy - RRMS	-3.7690004	0.0001639	0.00065561	DAG	18
OND - RRMS	-3.954758	7.6612E-05	0.00045967	DAG	18
CPMS - healthy	1.28043295	0.20039291	1	HexCer	34
CPMS - OND	1.24345294	0.21370097	0.85480389	HexCer	34
healthy - OND	-0.03698	0.97050094	0.97050094	HexCer	34
CPMS - RRMS	1.95069569	0.05109326	0.30655954	HexCer	34
healthy - RRMS	0.67026274	0.50269032	1	HexCer	34
OND - RRMS	0.70724275	0.47941564	1	HexCer	34
CPMS - healthy	0.08471894	0.93248484	0.93248484	HexCer	40
CPMS - OND	0.33099493	0.74064832	1	HexCer	40
healthy - OND	0.24627599	0.8054686	1	HexCer	40
CPMS - RRMS	1.17621411	0.23950933	1	HexCer	40
healthy - RRMS	1.09149517	0.27505506	1	HexCer	40
OND - RRMS	0.84521918	0.39798847	1	HexCer	40
CPMS - healthy	2.32810533	0.01990651	0.11943907	HexCer	42
CPMS - OND	2.16906325	0.03007788	0.15038941	HexCer	42
healthy - OND	-0.1590421	0.87363572	0.87363572	HexCer	42
CPMS - RRMS	1.91053528	0.05606433	0.22425731	HexCer	42
healthy - RRMS	-0.4175701	0.67626149	1	HexCer	42
OND - RRMS	-0.258528	0.79599946	1	HexCer	42
CPMS - healthy	-0.0711308	0.94329363	0.94329363	LPC	14
CPMS - OND	1.31030481	0.19009274	0.76037097	LPC	14
healthy - OND	1.38143564	0.16714505	0.83572527	LPC	14
CPMS - RRMS	-0.5353531	0.59240571	1	LPC	14
healthy - RRMS	-0.4642223	0.6424885	1	LPC	14
OND - RRMS	-1.8456579	0.06494189	0.38965135	LPC	14
CPMS - healthy	-0.6830799	0.49455636	1	LPC	16
CPMS - OND	0.79090002	0.42900234	1	LPC	16
healthy - OND	1.47397991	0.140487	0.842922	LPC	16
CPMS - RRMS	-0.3234604	0.74634663	0.74634663	LPC	16
healthy - RRMS	0.35961954	0.71913167	1	LPC	16
OND - RRMS	-1.1143604	0.26512462	1	LPC	16
CPMS - healthy	-2.2897373	0.02203655	0.13221931	LPC	18
CPMS - OND	-0.7178535	0.47284765	0.9456953	LPC	18
healthy - OND	1.5718838	0.1159775	0.46391001	LPC	18
CPMS - RRMS	-1.9041367	0.05689238	0.28446191	LPC	18
healthy - RRMS	0.38560055	0.69979252	0.69979252	LPC	18
OND - RRMS	-1.1862833	0.23551046	0.70653138	LPC	18
CPMS - healthy	0.92758245	0.35362421	1	LPC	20
CPMS - OND	1.38156759	0.16710451	1	LPC	20
healthy - OND	0.45398515	0.64983952	1	LPC	20
CPMS - RRMS	0.26803283	0.78867405	0.78867405	LPC	20
healthy - RRMS	-0.6595496	0.50954289	1	LPC	20

OND - RRMS	-1.1135348	0.26547883	1	LPC	20
CPMS - healthy	2.87453331	0.00404625	0.02023125	LPC	22
CPMS - OND	4.15122802	3.307E-05	0.00019842	LPC	22
healthy - OND	1.27669471	0.20171005	0.20171005	LPC	22
CPMS - RRMS	1.32792012	0.1842045	0.368409	LPC	22
healthy - RRMS	-1.5466132	0.12195655	0.36586964	LPC	22
OND - RRMS	-2.8233079	0.00475309	0.01901236	LPC	22
CPMS - healthy	-1.7925811	0.07303993	0.36519963	LPE	16
CPMS - OND	1.08371364	0.27849178	0.55698357	LPE	16
healthy - OND	2.87629477	0.00402374	0.02414243	LPE	16
CPMS - RRMS	-0.1521653	0.87905658	0.87905658	LPE	16
healthy - RRMS	1.64041586	0.10091873	0.40367492	LPE	16
OND - RRMS	-1.2358789	0.21650358	0.64951073	LPE	16
CPMS - healthy	-3.6763833	0.00023656	0.00141938	LPE	18
CPMS - OND	-0.4562354	0.64822073	1	LPE	18
healthy - OND	3.22014792	0.00128124	0.00640622	LPE	18
CPMS - RRMS	-0.5830072	0.55988845	1	LPE	18
healthy - RRMS	3.09337608	0.00197893	0.00791573	LPE	18
OND - RRMS	-0.1267718	0.89912099	0.89912099	LPE	18
CPMS - healthy	-1.3138094	0.18891036	0.56673109	LPE	20
CPMS - OND	1.33430852	0.18210277	0.72841106	LPE	20
healthy - OND	2.64811789	0.00809413	0.04856478	LPE	20
CPMS - RRMS	-0.2646254	0.79129803	0.79129803	LPE	20
healthy - RRMS	1.04918394	0.29409347	0.58818694	LPE	20
OND - RRMS	-1.598934	0.10983528	0.54917639	LPE	20
CPMS - healthy	0.13732083	0.8907772	0.8907772	LPE	22
CPMS - OND	2.76868491	0.0056283	0.03376982	LPE	22
healthy - OND	2.63136407	0.00850429	0.04252144	LPE	22
CPMS - RRMS	2.45321813	0.01415845	0.05663378	LPE	22
healthy - RRMS	2.3158973	0.02056388	0.06169163	LPE	22
OND - RRMS	-0.3154668	0.75240727	1	LPE	22
CPMS - healthy	-2.8552126	0.0043008	0.02150401	PC	14
CPMS - OND	-2.0457891	0.04077713	0.12233138	PC	14
healthy - OND	0.80942355	0.41827156	0.83654312	PC	14
CPMS - RRMS	0.02537696	0.97975429	0.97975429	PC	14
healthy - RRMS	2.88058961	0.00396932	0.02381593	PC	14
OND - RRMS	2.07116606	0.03834328	0.15337312	PC	14
CPMS - healthy	-5.5034589	3.7241E-08	1.8621E-07	PC	15
CPMS - OND	-3.6137777	0.00030177	0.0009053	PC	15
healthy - OND	1.88968119	0.05880061	0.11760122	PC	15
CPMS - RRMS	1.32698125	0.18451489	0.18451489	PC	15
healthy - RRMS	6.83044014	8.4654E-12	5.0793E-11	PC	15
OND - RRMS	4.94075895	7.7819E-07	3.1128E-06	PC	15
CPMS - healthy	-2.3218266	0.02024227	0.10121137	PC	16
CPMS - OND	-1.7286929	0.08386407	0.25159221	PC	16
healthy - OND	0.5931337	0.55309168	1	PC	16
CPMS - RRMS	0.0573492	0.95426703	0.95426703	PC	16

healthy - RRMS	2.37917583	0.0173514	0.10410838	PC	16
OND - RRMS	1.78604213	0.07409244	0.29636975	PC	16
CPMS - healthy	-6.1341501	8.5616E-10	3.4246E-09	PC	17
CPMS - OND	-5.7134628	1.107E-08	3.321E-08	PC	17
healthy - OND	0.4206873	0.67398343	0.67398343	PC	17
CPMS - RRMS	1.70544913	0.08811069	0.17622138	PC	17
healthy - RRMS	7.83959923	4.5199E-15	2.7119E-14	PC	17
OND - RRMS	7.41891193	1.1809E-13	5.9043E-13	PC	17
CPMS - healthy	-4.3395004	1.4281E-05	7.1404E-05	PC	18
CPMS - OND	-2.8253453	0.00472297	0.01416891	PC	18
healthy - OND	1.51415513	0.1299865	0.259973	PC	18
CPMS - RRMS	0.0068085	0.99456764	0.99456764	PC	18
healthy - RRMS	4.34630889	1.3845E-05	8.3069E-05	PC	18
OND - RRMS	2.83215376	0.00462356	0.01849424	PC	18
CPMS - healthy	-9.6085278	7.3594E-22	4.4156E-21	PC	20
CPMS - OND	-7.4298027	1.0876E-13	4.3504E-13	PC	20
healthy - OND	2.17872508	0.0293521	0.05870419	PC	20
CPMS - RRMS	-0.4944768	0.62096945	0.62096945	PC	20
healthy - RRMS	9.11405091	7.9362E-20	3.9681E-19	PC	20
OND - RRMS	6.93532582	4.0529E-12	1.2159E-11	PC	20
CPMS - healthy	-0.9384934	0.34799092	0.69598185	PC O-	16
CPMS - OND	-1.4033688	0.16050689	0.48152068	PC O-	16
healthy - OND	-0.4648754	0.64202067	0.64202067	PC O-	16
CPMS - RRMS	1.50329612	0.1327627	0.5310508	PC O-	16
healthy - RRMS	2.44178949	0.01461467	0.07307333	PC O-	16
OND - RRMS	2.90666491	0.00365304	0.02191826	PC O-	16
CPMS - healthy	-0.6976374	0.48540396	1	PC O-	17
CPMS - OND	-1.8411313	0.06560232	0.39361394	PC O-	17
healthy - OND	-1.1434938	0.25283361	1	PC O-	17
CPMS - RRMS	-0.1407595	0.88805995	0.88805995	PC O-	17
healthy - RRMS	0.55687796	0.57761082	1	PC O-	17
OND - RRMS	1.70037179	0.08906102	0.44530508	PC O-	17
CPMS - healthy	-2.881595	0.00395668	0.01187004	PC O-	18
CPMS - OND	-3.9144655	9.0605E-05	0.00036242	PC O-	18
healthy - OND	-1.0328705	0.3016645	0.3016645	PC O-	18
CPMS - RRMS	1.36181375	0.17325667	0.34651334	PC O-	18
healthy - RRMS	4.24340878	2.2015E-05	0.00011007	PC O-	18
OND - RRMS	5.27627929	1.3183E-07	7.91E-07	PC O-	18
CPMS - healthy	-5.3469659	8.9441E-08	4.472E-07	PE	16
CPMS - OND	-5.4849595	4.1356E-08	2.4814E-07	PE	16
healthy - OND	-0.1379935	0.89024554	0.89024554	PE	16
CPMS - RRMS	-0.1750369	0.86105067	1	PE	16
healthy - RRMS	5.17192909	2.3169E-07	6.9507E-07	PE	16
OND - RRMS	5.30992261	1.0967E-07	4.3869E-07	PE	16
CPMS - healthy	-3.8197777	0.00013357	0.00066786	PE	17
CPMS - OND	-1.8222793	0.06841262	0.13682524	PE	17
healthy - OND	1.99749844	0.04577106	0.13731319	PE	17

CPMS - RRMS	0.33875704	0.73479276	0.73479276	PE	17
healthy - RRMS	4.15853475	3.203E-05	0.00019218	PE	17
OND - RRMS	2.16103632	0.03069253	0.12277014	PE	17
CPMS - healthy	-7.7226142	1.1397E-14	6.8381E-14	PE	18
CPMS - OND	-6.364674	1.957E-10	7.8282E-10	PE	18
healthy - OND	1.35794024	0.17448265	0.34896529	PE	18
CPMS - RRMS	-0.3704696	0.71103261	0.71103261	PE	18
healthy - RRMS	7.35214463	1.9505E-13	9.7526E-13	PE	18
OND - RRMS	5.99420439	2.0448E-09	6.1345E-09	PE	18
CPMS - healthy	-2.5464852	0.01088138	0.05440691	PE	20
CPMS - OND	-2.8462335	0.00442397	0.02654385	PE	20
healthy - OND	-0.2997483	0.76436918	0.76436918	PE	20
CPMS - RRMS	-0.3602479	0.71866175	1	PE	20
healthy - RRMS	2.1862373	0.02879825	0.08639475	PE	20
OND - RRMS	2.48598556	0.01291932	0.05167728	PE	20
CPMS - healthy	-1.3460482	0.17828698	0.53486094	PE O-	16
CPMS - OND	-6.484357	8.9111E-11	5.3467E-10	PE O-	16
healthy - OND	-5.1383089	2.7722E-07	1.1089E-06	PE O-	16
CPMS - RRMS	-0.174822	0.86121953	0.86121953	PE O-	16
healthy - RRMS	1.17122622	0.24150786	0.48301572	PE O-	16
OND - RRMS	6.30953508	2.7987E-10	1.3994E-09	PE O-	16
CPMS - healthy	0.51595897	0.60588306	0.60588306	PE O-	17
CPMS - OND	-2.4033702	0.01624473	0.06497893	PE O-	17
healthy - OND	-2.9193292	0.00350786	0.01753928	PE O-	17
CPMS - RRMS	1.23497277	0.21684064	0.65052192	PE O-	17
healthy - RRMS	0.71901379	0.47213242	0.94426484	PE O-	17
OND - RRMS	3.63834295	0.0002744	0.00164639	PE O-	17
CPMS - healthy	-1.6039253	0.10873052	0.10873052	PE O-	18
CPMS - OND	-7.1836482	6.7875E-13	3.3938E-12	PE O-	18
healthy - OND	-5.5797229	2.409E-08	9.6361E-08	PE O-	18
CPMS - RRMS	1.75187634	0.07979507	0.15959015	PE O-	18
healthy - RRMS	3.35580161	0.00079135	0.00237406	PE O-	18
OND - RRMS	8.9355245	4.0525E-19	2.4315E-18	PE O-	18
CPMS - healthy	-4.45834	8.2597E-06	4.9558E-05	PI	16
CPMS - OND	-4.1163315	3.8495E-05	0.00019248	PI	16
healthy - OND	0.34200849	0.7323445	0.7323445	PI	16
CPMS - RRMS	-0.8328813	0.40491166	0.80982331	PI	16
healthy - RRMS	3.62545865	0.00028845	0.0011538	PI	16
OND - RRMS	3.28345017	0.00102545	0.00307634	PI	16
CPMS - healthy	-4.5581199	5.1614E-06	3.0968E-05	PI	18
CPMS - OND	-3.119554	0.00181125	0.007245	PI	18
healthy - OND	1.43856594	0.15027354	0.30054709	PI	18
CPMS - RRMS	-0.0600433	0.95212113	0.95212113	PI	18
healthy - RRMS	4.49807657	6.8571E-06	3.4286E-05	PI	18
OND - RRMS	3.05951064	0.00221699	0.00665097	PI	18
CPMS - healthy	0.61294728	0.5399112	1	SM	32
CPMS - OND	-0.0706724	0.94365848	1	SM	32

healthy - OND	-0.6836197	0.49421535	1	SM	32
CPMS - RRMS	0.65371983	0.51329233	1	SM	32
healthy - RRMS	0.04077255	0.96747723	0.96747723	SM	32
OND - RRMS	0.72439224	0.46882496	1	SM	32
CPMS - healthy	0.44422165	0.65688234	1	SM	34
CPMS - OND	-0.0637941	0.94913421	0.94913421	SM	34
healthy - OND	-0.5080157	0.61144233	1	SM	34
CPMS - RRMS	0.65429806	0.5129198	1	SM	34
healthy - RRMS	0.21007641	0.83360803	1	SM	34
OND - RRMS	0.71809212	0.47270049	1	SM	34
CPMS - healthy	1.47291302	0.14077449	0.70387245	SM	36
CPMS - OND	-0.3401814	0.73371993	0.73371993	SM	36
healthy - OND	-1.8130944	0.06981726	0.41890357	SM	36
CPMS - RRMS	1.01547763	0.30987821	0.92963462	SM	36
healthy - RRMS	-0.4574354	0.64735813	1	SM	36
OND - RRMS	1.35565901	0.17520769	0.70083074	SM	36
CPMS - healthy	1.50027718	0.13354262	0.80125571	SM	38
CPMS - OND	0.35238763	0.72454758	0.72454758	SM	38
healthy - OND	-1.1478895	0.25101416	1	SM	38
CPMS - RRMS	0.90858153	0.36357106	1	SM	38
healthy - RRMS	-0.5916956	0.55405441	1	SM	38
OND - RRMS	0.5561939	0.57807831	1	SM	38
CPMS - healthy	0.98352965	0.32534683	0.97604049	SM	40
CPMS - OND	-0.6797926	0.49663582	0.99327165	SM	40
healthy - OND	-1.6633222	0.09624796	0.48123979	SM	40
CPMS - RRMS	1.30041554	0.19345859	0.77383435	SM	40
healthy - RRMS	0.31688589	0.75133019	0.75133019	SM	40
OND - RRMS	1.98020809	0.04768015	0.28608091	SM	40
CPMS - healthy	1.14000027	0.25428619	1	SM	42
CPMS - OND	0.95591718	0.33911408	1	SM	42
healthy - OND	-0.1840831	0.85394827	0.85394827	SM	42
CPMS - RRMS	1.52788964	0.12653994	0.75923965	SM	42
healthy - RRMS	0.38788937	0.69809791	1	SM	42
OND - RRMS	0.57197246	0.56734064	1	SM	42
CPMS - healthy	-1.3594406	0.17400702	0.87003509	TAG	34
CPMS - OND	-0.322961	0.74672476	0.74672476	TAG	34
healthy - OND	1.03647956	0.29997847	1	TAG	34
CPMS - RRMS	0.70100067	0.4833026	0.96660519	TAG	34
healthy - RRMS	2.06044126	0.03935638	0.23613826	TAG	34
OND - RRMS	1.02396169	0.30585337	0.9175601	TAG	34
CPMS - healthy	-1.503767	0.13264136	0.39792408	TAG	38
CPMS - OND	-1.6637081	0.09617079	0.38468314	TAG	38
healthy - OND	-0.159941	0.87292754	0.87292754	TAG	38
CPMS - RRMS	0.98134664	0.32642183	0.65284366	TAG	38
healthy - RRMS	2.48511368	0.01295101	0.06475504	TAG	38
OND - RRMS	2.64505469	0.00816777	0.04900663	TAG	38
CPMS - healthy	0.25234329	0.80077573	1	TAG	40

CPMS - OND	-0.0928159	0.9260498	0.9260498	TAG	40
healthy - OND	-0.3451592	0.72997468	1	TAG	40
CPMS - RRMS	1.6735871	0.09421178	0.47105888	TAG	40
healthy - RRMS	1.42124381	0.15524589	0.62098357	TAG	40
OND - RRMS	1.76640302	0.07732826	0.46396955	TAG	40
CPMS - healthy	0.4052297	0.68530868	1	TAG	42
CPMS - OND	-0.2096965	0.83390453	0.83390453	TAG	42
healthy - OND	-0.6149262	0.53860344	1	TAG	42
CPMS - RRMS	0.99733717	0.31860088	1	TAG	42
healthy - RRMS	0.59210747	0.55377862	1	TAG	42
OND - RRMS	1.2070337	0.22741916	1	TAG	42
CPMS - healthy	1.82060331	0.06866718	0.41200308	TAG	43
CPMS - OND	0.71290752	0.47590299	1	TAG	43
healthy - OND	-1.1076958	0.26799322	1	TAG	43
CPMS - RRMS	1.14202521	0.25344354	1	TAG	43
healthy - RRMS	-0.6785781	0.49740522	0.99481044	TAG	43
OND - RRMS	0.42911769	0.66783758	0.66783758	TAG	43
CPMS - healthy	-1.0695177	0.28483648	0.56967295	TAG	44
CPMS - OND	-2.4453394	0.01447159	0.07235795	TAG	44
healthy - OND	-1.3758217	0.16887684	0.50663053	TAG	44
CPMS - RRMS	0.99748892	0.31852725	0.31852725	TAG	44
healthy - RRMS	2.06700657	0.03873353	0.15493414	TAG	44
OND - RRMS	3.44282827	0.00057566	0.00345399	TAG	44
CPMS - healthy	1.63383627	0.10229323	0.6137594	TAG	45
CPMS - OND	0.27137983	0.78609891	0.78609891	TAG	45
healthy - OND	-1.3624564	0.17305388	0.86526941	TAG	45
CPMS - RRMS	0.60666823	0.54407111	1	TAG	45
healthy - RRMS	-1.027168	0.30434134	1	TAG	45
OND - RRMS	0.3352884	0.73740755	1	TAG	45
CPMS - healthy	-0.0075124	0.994006	0.994006	TAG	46
CPMS - OND	-1.2170147	0.22359861	1	TAG	46
healthy - OND	-1.2095023	0.22646993	0.90587972	TAG	46
CPMS - RRMS	0.56718897	0.5705858	1	TAG	46
healthy - RRMS	0.5747014	0.56549326	1	TAG	46
OND - RRMS	1.7842037	0.07439057	0.44634343	TAG	46
CPMS - healthy	2.29896791	0.02150676	0.12904057	TAG	47
CPMS - OND	0.02706943	0.97840436	0.97840436	TAG	47
healthy - OND	-2.2718985	0.02309264	0.11546321	TAG	47
CPMS - RRMS	0.62221015	0.53380369	1	TAG	47
healthy - RRMS	-1.6767578	0.09358986	0.37435945	TAG	47
OND - RRMS	0.59514072	0.55174941	1	TAG	47
CPMS - healthy	-1.0565304	0.29072597	0.87217791	TAG	48
CPMS - OND	-2.2831761	0.02242	0.13451998	TAG	48
healthy - OND	-1.2266457	0.21995577	0.87982309	TAG	48
CPMS - RRMS	-0.5801608	0.56180616	1	TAG	48
healthy - RRMS	0.47636953	0.63381114	0.63381114	TAG	48
OND - RRMS	1.70301527	0.08856521	0.44282606	TAG	48

CPMS - healthy	0.07067849	0.94365364	1	TAG	49
CPMS - OND	-0.4213526	0.67349765	1	TAG	49
healthy - OND	-0.4920311	0.62269739	1	TAG	49
CPMS - RRMS	0.12980377	0.89672168	1	TAG	49
healthy - RRMS	0.05912528	0.95285232	0.95285232	TAG	49
OND - RRMS	0.55115634	0.58152651	1	TAG	49
CPMS - healthy	-0.566393	0.57112664	1	TAG	50
CPMS - OND	-0.8051815	0.42071497	1	TAG	50
healthy - OND	-0.2387885	0.81126962	1	TAG	50
CPMS - RRMS	-0.6823615	0.49501041	1	TAG	50
healthy - RRMS	-0.1159685	0.90767754	0.90767754	TAG	50
OND - RRMS	0.12281999	0.90224964	1	TAG	50
CPMS - healthy	-1.4423188	0.14921247	0.89527479	TAG	51
CPMS - OND	-1.3194494	0.18701891	0.93509455	TAG	51
healthy - OND	0.12286936	0.90221055	0.90221055	TAG	51
CPMS - RRMS	-0.1535867	0.87793562	1	TAG	51
healthy - RRMS	1.28873207	0.19749125	0.78996499	TAG	51
OND - RRMS	1.16586271	0.24366996	0.73100987	TAG	51
CPMS - healthy	-1.4685352	0.14195891	0.85175349	TAG	52
CPMS - OND	-1.1127164	0.26583023	1	TAG	52
healthy - OND	0.35581871	0.72197633	1	TAG	52
CPMS - RRMS	-1.0985726	0.27195454	1	TAG	52
healthy - RRMS	0.36996256	0.71141039	1	TAG	52
OND - RRMS	0.01414384	0.98871522	0.98871522	TAG	52
CPMS - healthy	-0.8662556	0.38635	1	TAG	53
CPMS - OND	-0.3295392	0.74174818	1	TAG	53
healthy - OND	0.53671643	0.59146351	1	TAG	53
CPMS - RRMS	0.14851054	0.88193987	0.88193987	TAG	53
healthy - RRMS	1.01476616	0.31021731	1	TAG	53
OND - RRMS	0.47804973	0.63261481	1	TAG	53
CPMS - healthy	-1.9638792	0.04954411	0.29726464	TAG	54
CPMS - OND	-1.3342714	0.18211493	0.72845971	TAG	54
healthy - OND	0.62960776	0.52895124	1	TAG	54
CPMS - RRMS	-0.1620412	0.87127344	0.87127344	TAG	54
healthy - RRMS	1.80183801	0.0715709	0.35785448	TAG	54
OND - RRMS	1.17223024	0.24110463	0.72331389	TAG	54
CPMS - healthy	0.89471297	0.37094054	0.74188109	TAG	55
CPMS - OND	-0.3653791	0.71482845	0.71482845	TAG	55
healthy - OND	-1.2600921	0.20763616	0.62290847	TAG	55
CPMS - RRMS	2.40694765	0.01608648	0.08043238	TAG	55
healthy - RRMS	1.51223467	0.13047417	0.52189669	TAG	55
OND - RRMS	2.77232673	0.00556571	0.03339428	TAG	55
CPMS - healthy	-1.4328121	0.15191154	0.60764616	TAG	56
CPMS - OND	-1.2336295	0.21734101	0.65202303	TAG	56
healthy - OND	0.19918264	0.84211988	0.84211988	TAG	56
CPMS - RRMS	0.83069004	0.40614875	0.81229751	TAG	56
healthy - RRMS	2.26350215	0.02360475	0.14162852	TAG	56

OND - RRMS	2.0643195	0.03898743	0.19493717	TAG	56
CPMS - healthy	-1.4426378	0.14912254	0.7456127	TAG	57
CPMS - OND	-1.033793	0.30123292	0.90369876	TAG	57
healthy - OND	0.40884471	0.68265363	1	TAG	57
CPMS - RRMS	0.39716343	0.69124695	0.69124695	TAG	57
healthy - RRMS	1.83980119	0.06579743	0.39478458	TAG	57
OND - RRMS	1.43095648	0.15244269	0.60977075	TAG	57
CPMS - healthy	-3.6689366	0.00024356	0.00121781	TAG	58
CPMS - OND	-1.6016519	0.10923261	0.21846521	TAG	58
healthy - OND	2.06728465	0.03870734	0.15482936	TAG	58
CPMS - RRMS	0.22900539	0.81886473	0.81886473	TAG	58
healthy - RRMS	3.89794194	9.7014E-05	0.00058208	TAG	58
OND - RRMS	1.83065729	0.06715171	0.20145513	TAG	58

This table reports the results of the Kruskal-Wallis test with Dunn post hoc test (P.unadj) and Holm correction (P.adj) for the analysis of the first chain length.

Cholesteryl ester (CE), Ceramide (Cer), Cholesterol (Chol), Diacylglycerol (DAG), Hexosylceramide (HexCer), Lysophosphatidylcholine (LPC), Ether-linked Lysophosphatidylcholine (LPC O-), Lysophosphatidylethanolamine (LPE), Ether-linked Lysophosphatidylethanolamine (LPE O-), Phosphatidylcholine (PC), Ether-linked Phosphatidylcholine (PC O-), Phosphatidylethanolamine (PE), Ether-linked Phosphatidylethanolamine (PE O-) Phosphatidylinositol (PI), Sphingomyelin (SM), Triacylglycerol (TAG).

Chain-Length Analysis- Chain 2

Supplement Table S5 Chain-Length Analysis Part 2

Comparison	Z	P.unadj	P.adj	Class	Chain
CPMS - healthy	6.32948118	2.4599E-10	1.2299E-09	DAG	18
CPMS - OND	5.78555208	7.2275E-09	2.1682E-08	DAG	18
healthy - OND	-0.5439291	0.58649026	1	DAG	18
CPMS - RRMS	-0.1723845	0.8631353	0.8631353	DAG	18
healthy - RRMS	-6.5018656	7.933E-11	4.7598E-10	DAG	18
OND - RRMS	-5.9579365	2.5544E-09	1.0218E-08	DAG	18
CPMS - healthy	4.11991117	3.7902E-05	0.00022741	DAG	16
CPMS - OND	3.74654422	0.00017929	0.00089644	DAG	16
healthy - OND	-0.373367	0.70887535	0.70887535	DAG	16
CPMS - RRMS	0.57936251	0.5623446	1	DAG	16
healthy - RRMS	-3.5405487	0.0003993	0.00159718	DAG	16
OND - RRMS	-3.1671817	0.00153924	0.00461772	DAG	16
CPMS - healthy	4.64651286	3.3759E-06	2.0256E-05	DAG	17
CPMS - OND	4.2629054	2.0179E-05	0.00010089	DAG	17
healthy - OND	-0.3836075	0.70126941	0.70126941	DAG	17
CPMS - RRMS	2.22060091	0.026378	0.07913401	DAG	17
healthy - RRMS	-2.4259119	0.01526997	0.0610799	DAG	17
OND - RRMS	-2.0423045	0.04112134	0.08224267	DAG	17
CPMS - healthy	-3.1721984	0.0015129	0.00605158	DAG	20
CPMS - OND	-4.5123519	6.4113E-06	3.8468E-05	DAG	20
healthy - OND	-1.3401534	0.18019546	0.36039093	DAG	20
CPMS - RRMS	-0.3001542	0.76405952	0.76405952	DAG	20
healthy - RRMS	2.8720442	0.00407826	0.01223478	DAG	20
OND - RRMS	4.21219765	2.529E-05	0.00012645	DAG	20
CPMS - healthy	-3.8186771	0.00013417	0.00067085	PC	17
CPMS - OND	-2.5385025	0.0111328	0.0333984	PC	17
healthy - OND	1.28017464	0.20048372	0.40096744	PC	17
CPMS - RRMS	0.439548	0.66026451	0.66026451	PC	17
healthy - RRMS	4.25822514	2.0606E-05	0.00012363	PC	17
OND - RRMS	2.97805049	0.00290088	0.01160353	PC	17
CPMS - healthy	-2.6227214	0.00872306	0.04361528	PC	18
CPMS - OND	-1.4492349	0.14727199	0.44181596	PC	18
healthy - OND	1.17348651	0.24060076	0.48120153	PC	18
CPMS - RRMS	0.3687395	0.7123219	0.7123219	PC	18
healthy - RRMS	2.99146094	0.00277646	0.01665876	PC	18
OND - RRMS	1.81797443	0.06906804	0.27627216	PC	18
CPMS - healthy	-7.2877957	3.1507E-13	1.5753E-12	PC	20
CPMS - OND	-5.7561511	8.6053E-09	2.5816E-08	PC	20
healthy - OND	1.53164459	0.12561016	0.25122032	PC	20
CPMS - RRMS	0.10872293	0.91342225	0.91342225	PC	20
healthy - RRMS	7.39651865	1.398E-13	8.3881E-13	PC	20
OND - RRMS	5.86487406	4.4947E-09	1.7979E-08	PC	20

CPMS - healthy	-3.5304774	0.00041481	0.00165924	PC	15
CPMS - OND	-2.7387159	0.00616796	0.01850389	PC	15
healthy - OND	0.79176147	0.42849977	0.42849977	PC	15
CPMS - RRMS	1.63111517	0.10286602	0.20573204	PC	15
healthy - RRMS	5.16159256	2.4486E-07	1.4691E-06	PC	15
OND - RRMS	4.36983109	1.2434E-05	6.2171E-05	PC	15
CPMS - healthy	-2.8156122	0.00486844	0.01947376	PC	22
CPMS - OND	-2.4991812	0.01244807	0.0373442	PC	22
healthy - OND	0.31643105	0.75167535	0.75167535	PC	22
CPMS - RRMS	0.72248425	0.46999681	0.93999361	PC	22
healthy - RRMS	3.53809646	0.00040302	0.00241814	PC	22
OND - RRMS	3.22166541	0.00127448	0.00637239	PC	22
CPMS - healthy	-6.1159534	9.5981E-10	4.7991E-09	PC	19
CPMS - OND	-3.7833189	0.00015475	0.00046425	PC	19
healthy - OND	2.33263452	0.01966734	0.03933467	PC	19
CPMS - RRMS	1.162196	0.24515586	0.24515586	PC	19
healthy - RRMS	7.27814939	3.3843E-13	2.0306E-12	PC	19
OND - RRMS	4.94551488	7.5943E-07	3.0377E-06	PC	19
CPMS - healthy	-4.2742001	1.9182E-05	0.00011509	PC	24
CPMS - OND	-2.2437012	0.02485163	0.09940653	PC	24
healthy - OND	2.03049884	0.04230586	0.12691758	PC	24
CPMS - RRMS	-0.2639648	0.791807	0.791807	PC	24
healthy - RRMS	4.01023521	6.0658E-05	0.00030329	PC	24
OND - RRMS	1.97973637	0.04773316	0.09546632	PC	24
CPMS - healthy	-1.0305732	0.30274101	0.60548203	PC O-	20
CPMS - OND	-2.6662525	0.00767021	0.03835103	PC O-	20
healthy - OND	-1.6356793	0.10190672	0.40762689	PC O-	20
CPMS - RRMS	0.59452528	0.55216084	0.55216084	PC O-	20
healthy - RRMS	1.62509847	0.10414158	0.31242473	PC O-	20
OND - RRMS	3.26077778	0.00111107	0.00666642	PC O-	20
CPMS - healthy	-4.1482479	3.3503E-05	0.00010051	PC O-	22
CPMS - OND	-5.6379678	1.7207E-08	6.8827E-08	PC O-	22
healthy - OND	-1.4897199	0.13629789	0.13629789	PC O-	22
CPMS - RRMS	**2.51141852**	**0.0120247**	**0.02404941**	**PC O-**	**22**
healthy - RRMS	6.6596664	2.7445E-11	1.3722E-10	PC O-	22
OND - RRMS	8.14938632	3.6578E-16	2.1947E-15	PC O-	22
CPMS - healthy	-1.5253667	0.12716766	0.50867062	PC O-	15
CPMS - OND	-0.115052	0.90840391	0.90840391	PC O-	15
healthy - OND	1.41031472	0.15844677	0.31689355	PC O-	15
CPMS - RRMS	1.47711911	0.13964371	0.41893114	PC O-	15
healthy - RRMS	3.00248582	0.00267784	0.01606707	PC O-	15
OND - RRMS	1.5921711	0.11134627	0.55673133	PC O-	15
CPMS - healthy	-3.2601043	0.00111371	0.00668228	PC O-	17
CPMS - OND	-2.8683654	0.00412599	0.02062994	PC O-	17
healthy - OND	0.39173888	0.69525116	0.69525116	PC O-	17
CPMS - RRMS	-0.8087839	0.41863946	0.83727893	PC O-	17

healthy - RRMS	2.45132039	0.01423332	0.05693328	PC O-	17
OND - RRMS	2.05958151	0.03943857	0.1183157	PC O-	17
CPMS - healthy	-3.7164913	0.00020201	0.00080803	PC O-	19
CPMS - OND	-3.6517763	0.00026043	0.0007813	PC O-	19
healthy - OND	0.06471502	0.9484009	0.9484009	PC O-	19
CPMS - RRMS	0.32357511	0.74625973	1	PC O-	19
healthy - RRMS	4.04006643	5.3436E-05	0.00032062	PC O-	19
OND - RRMS	3.97535141	7.0275E-05	0.00035138	PC O-	19
CPMS - healthy	-4.8381051	1.3108E-06	7.865E-06	PE	18
CPMS - OND	-2.9402888	0.00327906	0.01311626	PE	18
healthy - OND	1.89781622	0.0577203	0.11544059	PE	18
CPMS - RRMS	-0.8628902	0.38819783	0.38819783	PE	18
healthy - RRMS	3.97521483	7.0316E-05	0.00035158	PE	18
OND - RRMS	2.07739861	0.03776478	0.11329435	PE	18
CPMS - healthy	-4.94761	7.513E-07	4.5078E-06	PE	20
CPMS - OND	-3.9328408	8.3948E-05	0.00033579	PE	20
healthy - OND	1.01476915	0.31021588	0.62043176	PE	20
CPMS - RRMS	-0.0836146	0.93336285	0.93336285	PE	20
healthy - RRMS	4.86399539	1.1504E-06	5.752E-06	PE	20
OND - RRMS	3.84922623	0.00011849	0.00035547	PE	20
CPMS - healthy	-7.6005624	2.9485E-14	8.8454E-14	PE	22
CPMS - OND	-8.8124734	1.2241E-18	6.1207E-18	PE	22
healthy - OND	-1.211911	0.22554646	0.45109292	PE	22
CPMS - RRMS	0.43824549	0.66120834	0.66120834	PE	22
healthy - RRMS	8.03880788	9.0717E-16	3.6287E-15	PE	22
OND - RRMS	9.25071886	2.2299E-20	1.3379E-19	PE	22
CPMS - healthy	1.1714619	0.24141316	0.72423949	PE O-	18
CPMS - OND	-2.2346309	0.02544159	0.10176635	PE O-	18
healthy - OND	-3.4060928	0.000659	0.00395399	PE O-	18
CPMS - RRMS	0.3893924	0.6969859	0.6969859	PE O-	18
healthy - RRMS	-0.7820695	0.43417373	0.86834746	PE O-	18
OND - RRMS	2.62402333	0.00868978	0.04344892	PE O-	18
CPMS - healthy	0.37869678	0.70491304	0.70491304	PE O-	20
CPMS - OND	-4.9171254	8.7824E-07	3.513E-06	PE O-	20
healthy - OND	-5.2958222	1.1848E-07	5.9241E-07	PE O-	20
CPMS - RRMS	1.3091463	0.1904848	0.5714544	PE O-	20
healthy - RRMS	0.93044951	0.35213839	0.70427679	PE O-	20
OND - RRMS	6.22627167	4.7767E-10	2.866E-09	PE O-	20
CPMS - healthy	-4.8427586	1.2805E-06	2.561E-06	PE O-	22
CPMS - OND	-9.7241633	2.3785E-22	1.1893E-21	PE O-	22
healthy - OND	-4.8814046	1.0533E-06	3.16E-06	PE O-	22
CPMS - RRMS	0.74444951	0.45660457	0.45660457	PE O-	22
healthy - RRMS	5.58720816	2.3075E-08	9.23E-08	PE O-	22
OND - RRMS	10.4686128	1.2039E-25	7.2237E-25	PE O-	22
CPMS - healthy	1.89735843	0.05778065	0.28890324	PE O-	16
CPMS - OND	-1.7166233	0.08604802	0.17209603	PE O-	16

healthy - OND	-3.6139818	0.00030153	0.00180918	PE O-	16
CPMS - RRMS	0.10152848	0.91913095	0.91913095	PE O-	16
healthy - RRMS	-1.79583	0.07252157	0.2175647	PE O-	16
OND - RRMS	1.81815182	0.06904093	0.27616373	PE O-	16
CPMS - healthy	-5.4941189	3.9267E-08	2.356E-07	PI	18
CPMS - OND	-3.4866723	0.00048907	0.00195628	PI	18
healthy - OND	2.00744662	0.04470213	0.13410639	PI	18
CPMS - RRMS	-1.6369324	0.10164459	0.10164459	PI	18
healthy - RRMS	3.8571865	0.0001147	0.0005735	PI	18
OND - RRMS	1.84973988	0.06435105	0.1287021	PI	18
CPMS - healthy	-5.0372979	4.7215E-07	1.4164E-06	PI	22
CPMS - OND	-5.8063549	6.3848E-09	2.5539E-08	PI	22
healthy - OND	-0.7690571	0.44185944	0.44185944	PI	22
CPMS - RRMS	1.91206874	0.05586738	0.11173476	PI	22
healthy - RRMS	6.94936663	3.6693E-12	1.8346E-11	PI	22
OND - RRMS	7.71842368	1.1778E-14	7.0666E-14	PI	22

This table reports the results of the Kruskal-Wallis test with Dunn post hoc test (P.unadj) and Holm correction (P.adj) for the analysis of the second chain length.

Cholesteryl ester (CE), Ceramide (Cer), Cholesterol (Chol), Diacylglycerol (DAG), Hexosylceramide (HexCer), Lysophosphatidylcholine (LPC), Ether-linked Lysophosphatidylcholine (LPC O-), Lysophosphatidylethanolamine (LPE), Ether-linked Lysophosphatidylethanolamine (LPE O-), Phosphatidylcholine (PC), Ether-linked Phosphatidylcholine (PC O-), Phosphatidylethanolamine (PE), Ether-linked Phosphatidylethanolamine (PE O-) Phosphatidylinositol (PI), Sphingomyelin (SM), Triacylglycerol (TAG).

Supplement Table S6 Disease Modifying Therapy (DMT)

Disease	Disease Modifying Therapy	Activity
**CPMS**	-	inactive
**CPMS**	-	inactive
**CPMS**	-	active
**CPMS**	-	inactive
**CPMS**	-	inactive
**CPMS**	Dimethylfumarat	inactive
**CPMS**	Dimethylfumarat	inactive
**CPMS**	Fingolimod	inactive
**CPMS**	Glatirameracetat	inactive
**CPMS**	Methylprednisolon 1g/3d	inactive
**CPMS**	Methylprednisolon 1g/3d	inactive
**CPMS**	Methylprednisolon 1g/3d	inactive
**CPMS**	Methylprednisolon 1g/3d	inactive
**CPMS**	Methylprednisolon 1g/3d	inactive
**CPMS**	Methylprednisolon 1g/3d	inactive
**CPMS**	Methylprednisolon 1g/3d	inactive
**CPMS**	Methylprednisolon 1g/3d	inactive
**CPMS**	Methylprednisolon 1g/3d	inactive
**CPMS**	Methylprednisolon 1g/3d	inactive
**CPMS**	Methylprednisolon 1g/3d	active
**CPMS**	Methylprednisolon 1g/3d	inactive
**CPMS**	Methylprednisolon 1g/3d	inactive
**CPMS**	Methylprednisolon 1g/3d	inactive
**CPMS**	Methylprednisolon 1g/3d	inactive
**CPMS**	Methylprednisolon 1g/3d	inactive
**CPMS**	Methylprednisolon 1g/3d	inactive
**CPMS**	Ocrelizumab	inactive
**CPMS**	Ocrelizumab	inactive
**CPMS**	Ocrelizumab	inactive
**CPMS**	Rituximab	inactive
**RRMS**	-	active
**RRMS**	-	active
**RRMS**	-	active
**RRMS**	-	active
**RRMS**	-	active
**RRMS**	-	active
**RRMS**	-	active
**RRMS**	Fingolimod	active
**RRMS**	Fingolimod	inactive
**RRMS**	Glatirameracetat	inactive
**RRMS**	Glatirameracetat	active
**RRMS**	Natalizumab	inactive
**RRMS**	Natalizumab	inactive

**RRMS**	Natalizumab	inactive
**RRMS**	Natalizumab	inactive
**RRMS**	Natalizumab	inactive
**RRMS**	Natalizumab	inactive
**RRMS**	Natalizumab	inactive
**RRMS**	Natalizumab	inactive
**RRMS**	Natalizumab	active
**RRMS**	Natalizumab	inactive
**RRMS**	Natalizumab	inactive
**RRMS**	Natalizumab	inactive
**RRMS**	Natalizumab	inactive
**RRMS**	Natalizumab	inactive
**RRMS**	Ocrelizumab	inactive
**RRMS**	Ocrelizumab	inactive
**RRMS**	Ocrelizumab	active
**RRMS**	Ocrelizumab	inactive
**RRMS**	Ocrelizumab	inactive

Supplement- Figures

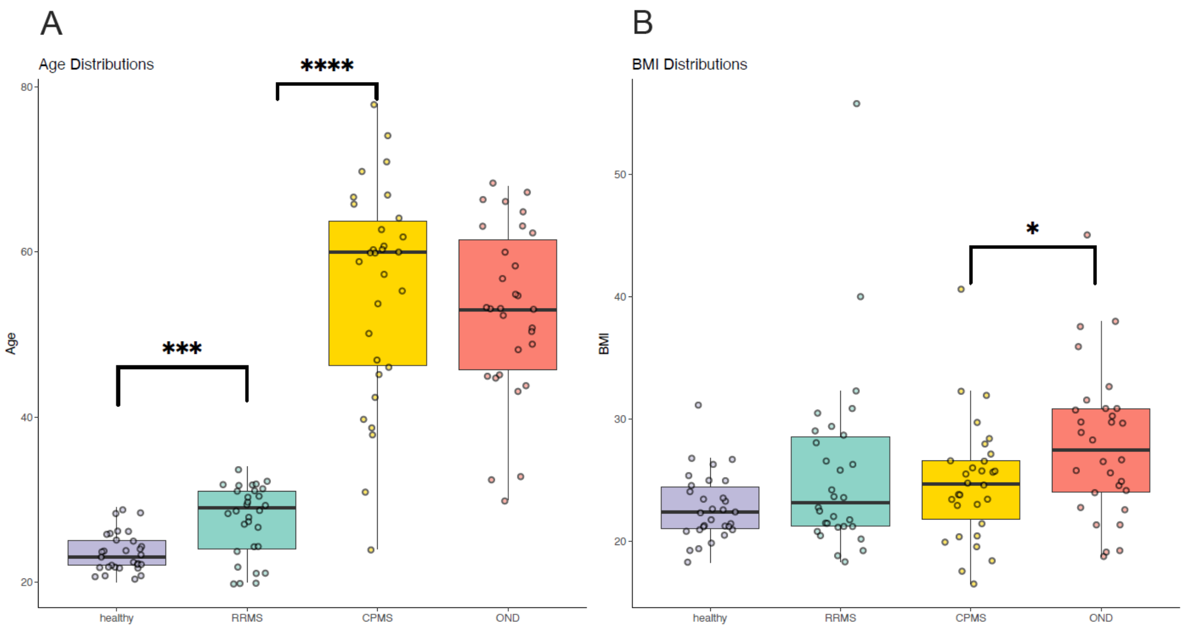

Supplement Figure S1 Distribution of Age and BMI

*Box plots visualizing the distribution of age (**A**) and BMI (**B**). For statistical evaluation. Significance was determined with MWU-test (Supplement Table 1 and 2). Only significant differences in the matched cohorts healthy vs RRMS and OND vs CPMS were highlighted; * indicates p < 0.05. *** indicates p < 0.001. **** indicates p < 0.0001.*

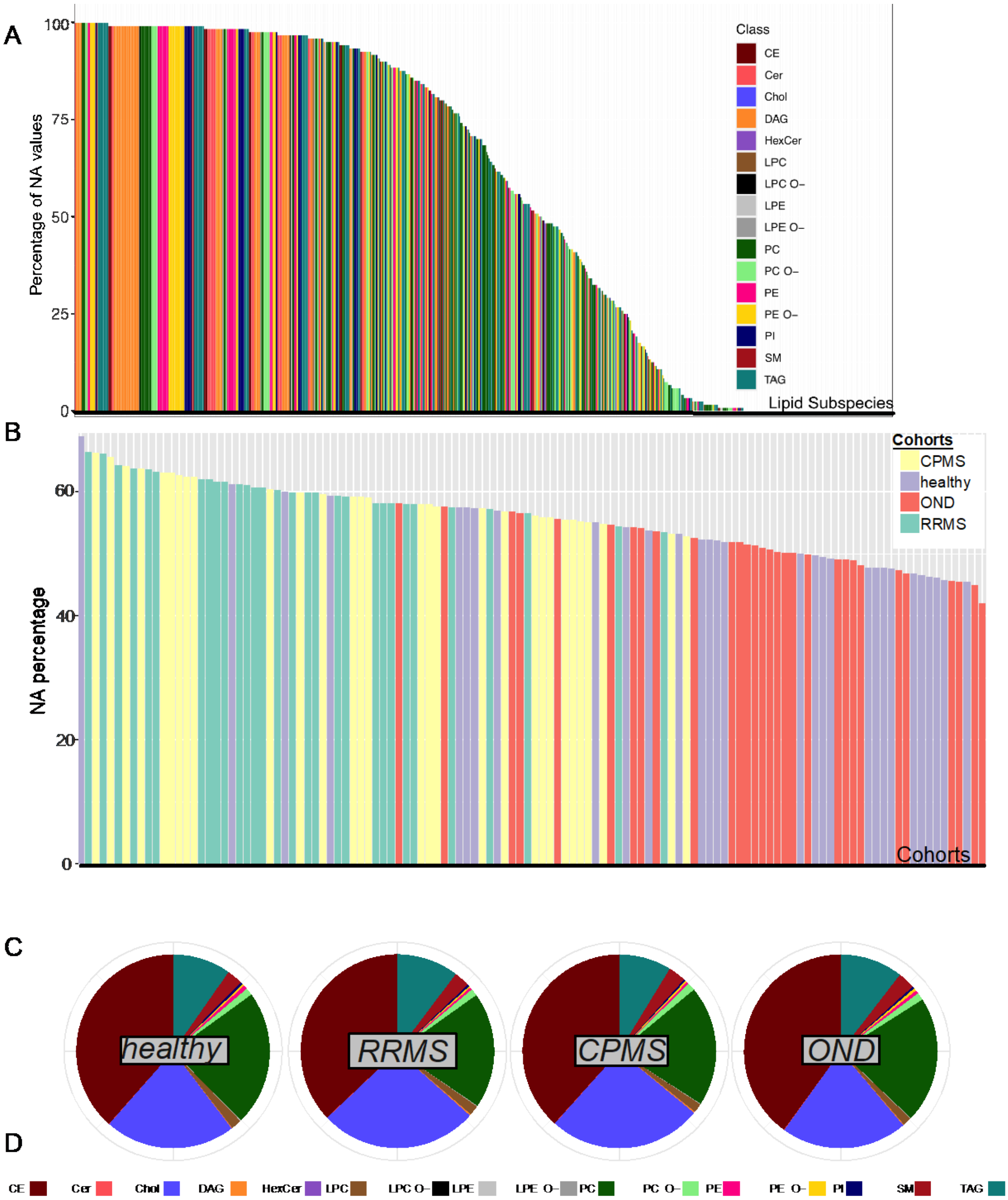

D

Distribution of Lipid Class [%]

cohort	CE	Cer	Chol	DAG	HexCer	LPC	LPC O-	LPE	LPE O-	PC	PC O-	PE	PE O-	PI	SM	TAG
CPMS	38.32	0.06	25.68	0.21	0.02	1.51	<0.00	0.11	<0.00	20.11	1.29	0.39	0.31	0.32	2.94	8.74
healthy	38.47	0.05	21.84	0.12	0.01	1.76	<0.00	0.13	<0.00	22.60	1.24	0.68	0.31	0.43	2.64	9.73
OND	40.01	0.06	21.11	0.15	0.01	1.44	<0.00	0.10	<0.00	21.11	1.27	0.56	0.47	0.36	2.88	10.47
RRMS	37.01	0.05	26.41	0.22	0.01	1.65	<0.00	0.11	<0.00	19.38	1.18	0.41	0.28	0.32	2.65	10.31

Supplement Figure S2 Data Imputation

*(**A**) shows the percentage of missing values of each lipid (color-coded according to the respective lipid class). (**B**) shows the percentage of missing data among the participants (color-coded according to the respective cohort). A slight accumulation of missing values in the DAG class as well as in the cohorts of RRMS and CPMS, in contrast to OND and healthy is observed. A 90% missing value filter was applied to remove lipids with predominantly missing data, followed by an imputation with zero , due to the assumption of left missingness. (**C**) shows the percentage distribution of lipid class in a pie chart with respect to each cohort. The corresponding percentages are listed in (**D**). Cholesteryl ester (CE), Ceramide (Cer), Cholesterol (Chol), Diacylglycerol (DAG), Hexosylceramide (HexCer), Lysophosphatidylcholine (LPC), Ether-linked Lysophosphatidylcholine (LPC O-), Lysophosphatidylethanolamine (LPE), Ether-linked Lysophosphatidylethanolamine (LPE O-), Phosphatidylcholine (PC), Ether-linked Phosphatidylcholine (PC O-), Phosphatidylethanolamine (PE), Ether-linked Phosphatidylethanolamine (PE O-) Phosphatidylinositol (PI), Sphingomyelin (SM), Triacylglycerol (TAG).*

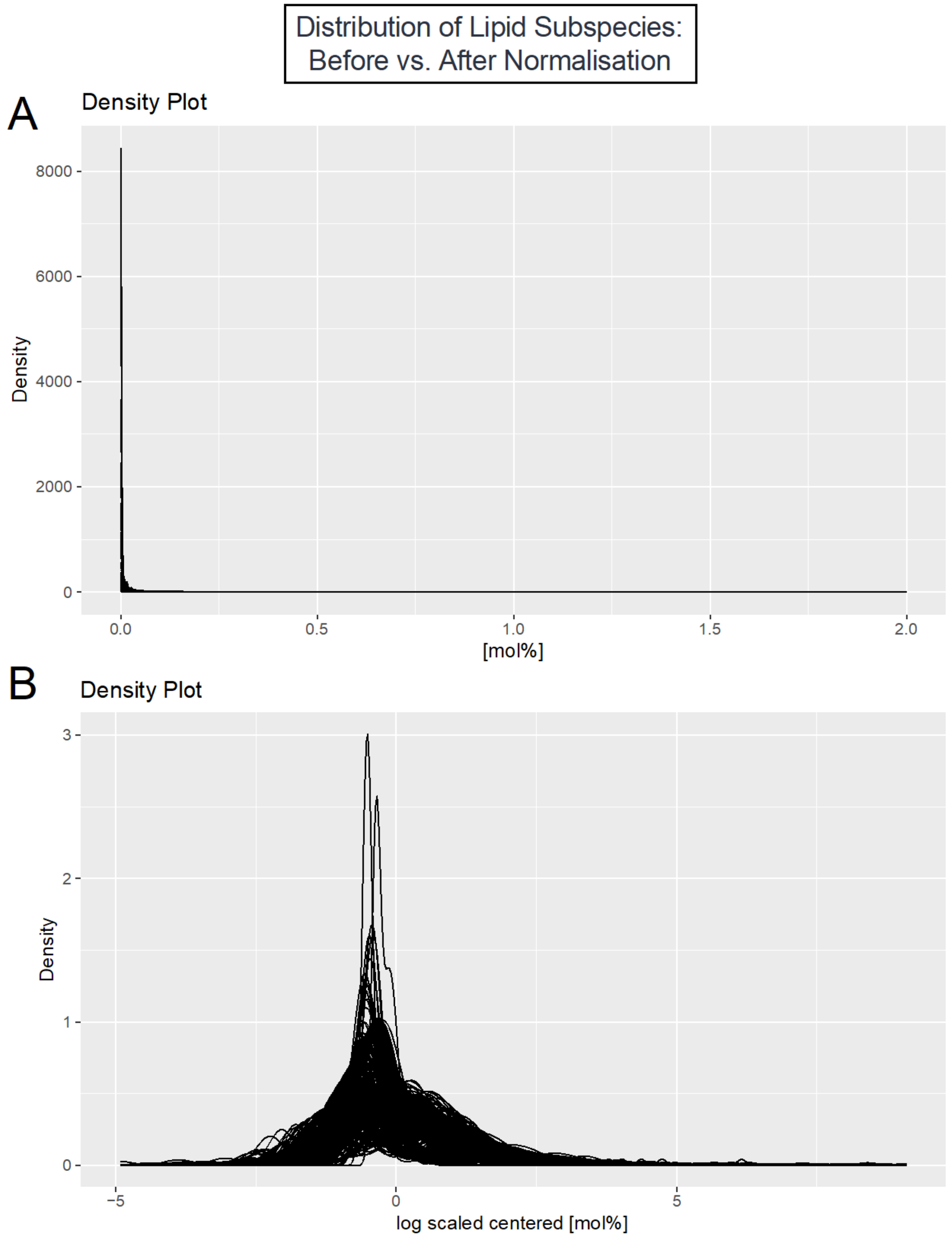

Supplement Figure S3 Distribution of Lipid Subspecies

(A) *Distribution without normalizing.* ***(B)*** *Distribution after normalizing by log transformation, centering and scaling resulted in an approximately normal distribution of lipid species. Therefore, statistical tests under the assumption of normal distribution have been used. Each lipid species is represented by a unique color within a continuous color scale. This color coding is designed to provide a macroscopic overview of the overall distribution patterns of the lipid measurements.*

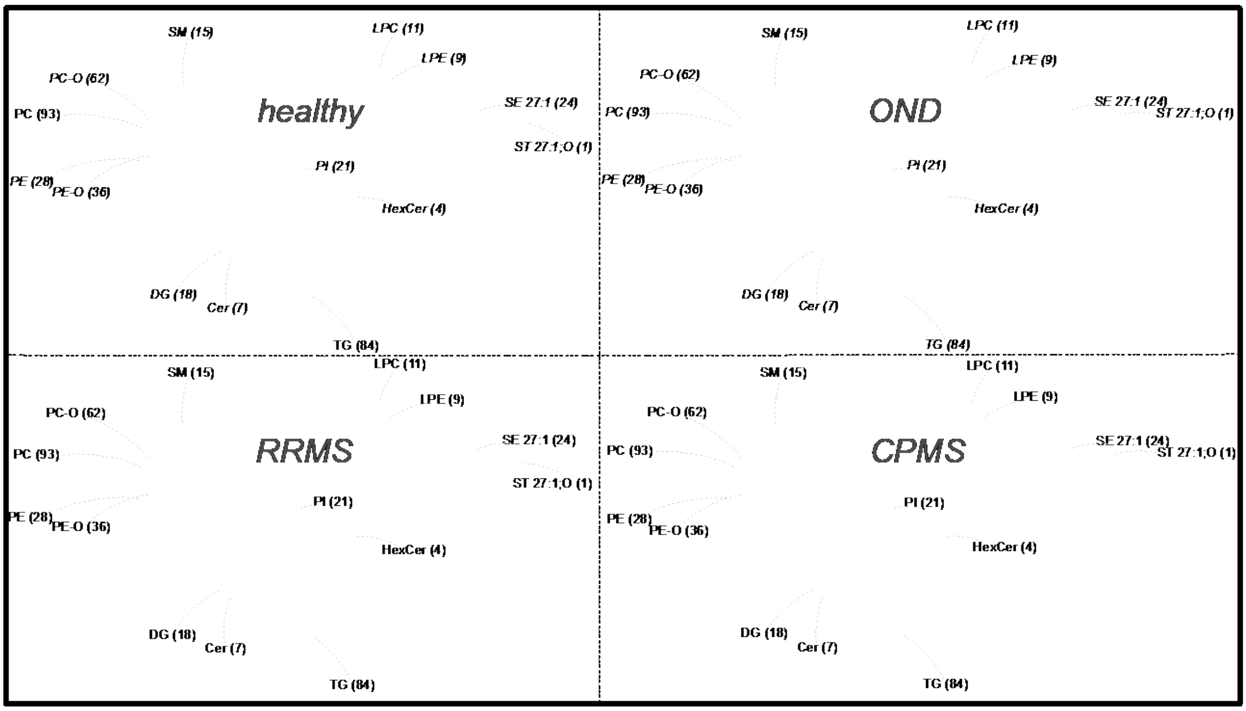

Supplement Figure S4 Lipid-Space-Chart

The lipid space plot was generated using LipidSpace from Lipidomics Informatics for Life Sciences [49]. For each cohort, all lipids are pairwise compared to each other, scored based on their similarity, and visualized by a dot. The calculated similarity defines the proximity of the individual dots. Quantity is visualized by the size of the dots. Prior Goslin [50] was leveraged to generate a nomenclature of lipids used in LipidSpace differing from the otherwise used nomenclature in this paper.

Cholesterol (ST), Steryl ester (SE), Ceramide (Cer), Diacylglycerol (DG), Hexosylceramide (HexCer), Lysophosphatidylcholine (LPC), Lysophosphatidylethanolamine (LPE), Phosphatidylcholine (PC), Ether-linked Phosphatidylcholine (PC O-), Phosphatidylethanolamine (PE), Ether-linked Phosphatidylethanolamine (PE -O) Phosphatidylinositol (PI), Sphingomyelin (SM), Triacylglycerol (TG).

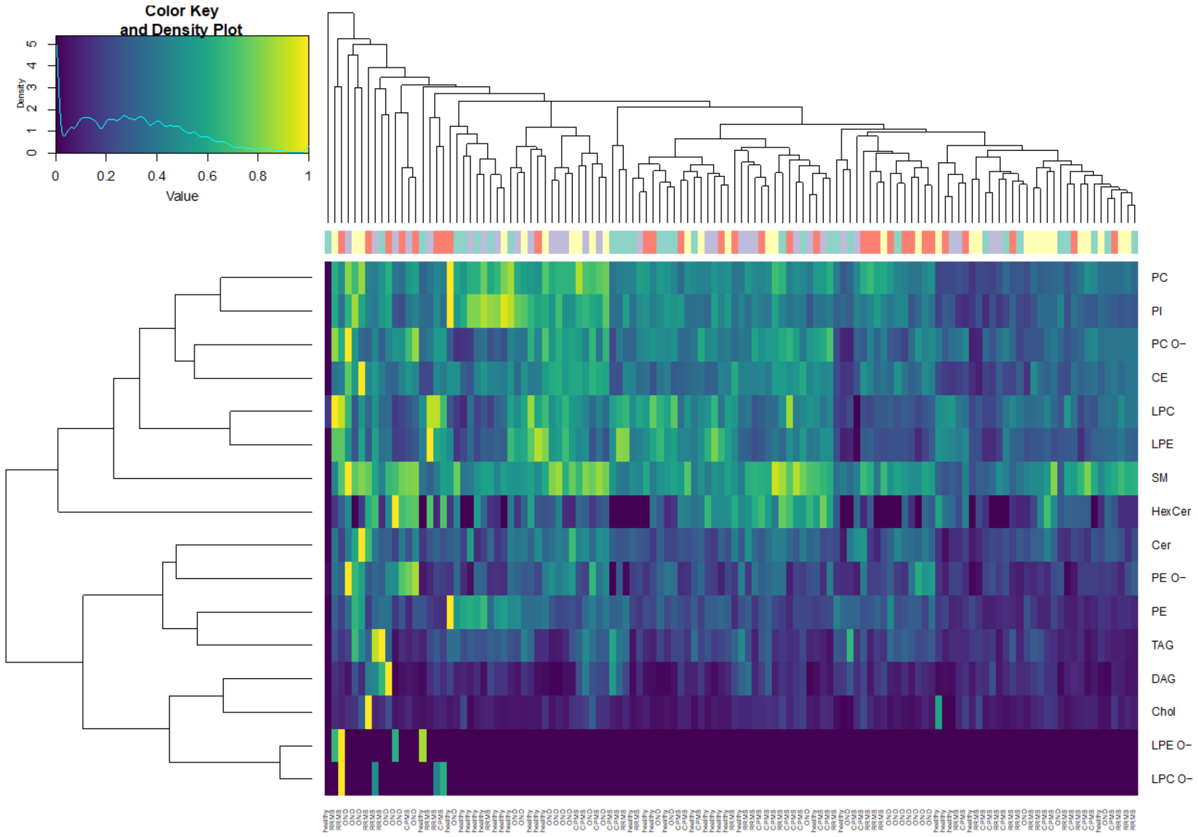

Supplement Figure S5 Heatmap with Hierarchical Clustering

Each lipid class was standardized using min-max normalization for heatmap generation. The heatmap's color coding at its top indicates cohort assignments. Hierarchical clustering is represented by dendrograms and was performed using the Manhattan distance metric and the average linkage method.

Cholesteryl ester (CE), Ceramide (Cer), Cholesterol (Chol), Diacylglycerol (DAG), Hexosylceramide (HexCer), Lysophosphatidylcholine (LPC), Ether-linked Lysophosphatidylcholine (LPC O-), Lysophosphatidylethanolamine (LPE), Ether-linked Lysophosphatidylethanolamine (LPE O-), Phosphatidylcholine (PC), Ether-linked Phosphatidylcholine (PC O-), Phosphatidylethanolamine (PE), Ether-linked Phosphatidylethanolamine (PE O-) Phosphatidylinositol (PI), Sphingomyelin (SM), Triacylglycerol (TAG).

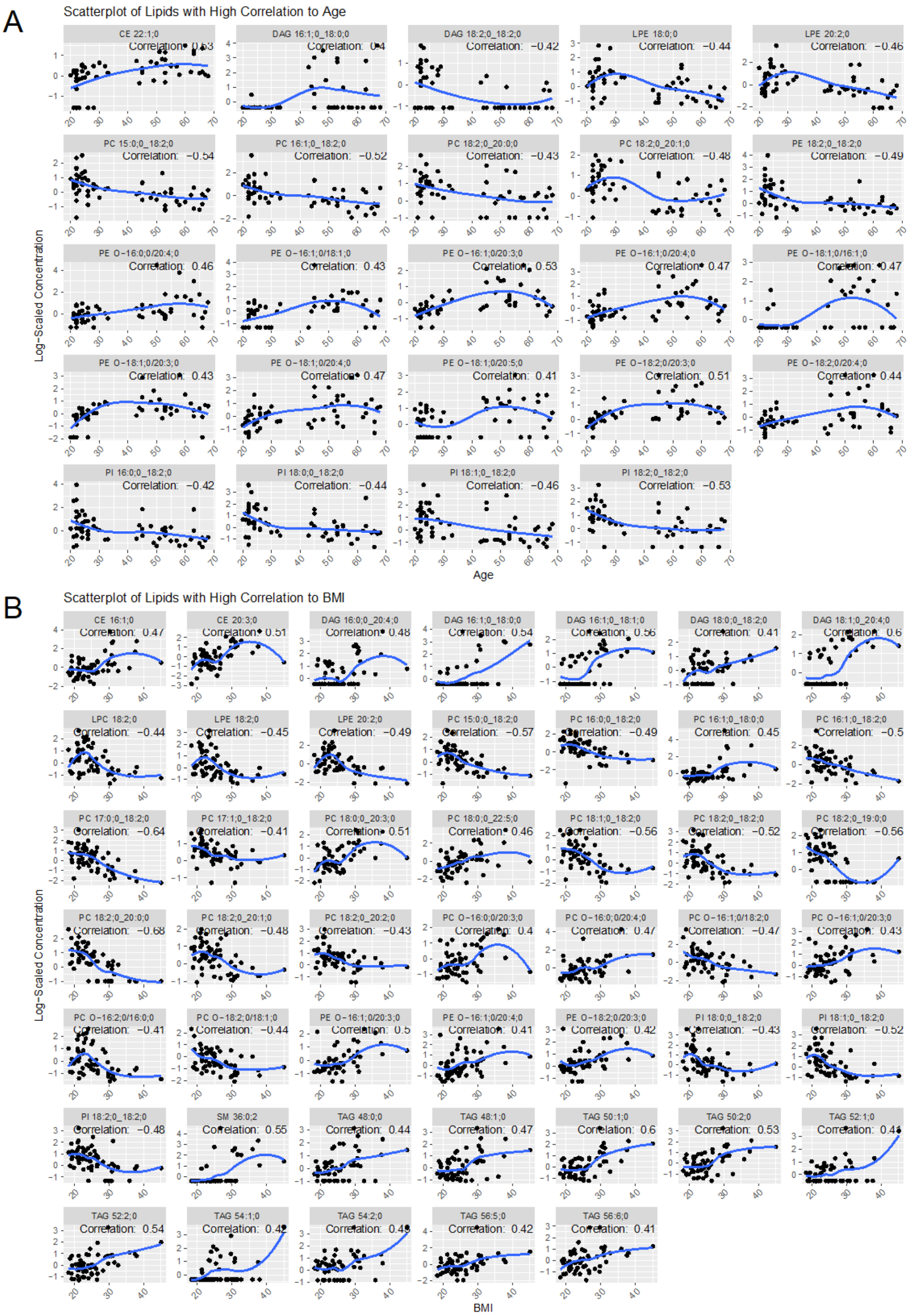

Supplement Figure S6 Scatter plots of Lipids with Correlation to Age and BMI

*In the presented scatter plots lipids that are correlated with age (**A**) and BMI (**B**) are visualized. Only lipids exhibiting an absolute Kendall tau > 0.4 are considered correlated and thus displayed. Additionally to visualize the trend a LOESS-smoothed line has been integrated into each plot.*

Cholesteryl ester (CE), Ceramide (Cer), Cholesterol (Chol), Diacylglycerol (DAG), Hexosylceramide (HexCer), Lysophosphatidylcholine (LPC), Ether-linked Lysophosphatidylcholine (LPC O-), Lysophosphatidylethanolamine (LPE), Ether-linked Lysophosphatidylethanolamine (LPE O-), Phosphatidylcholine (PC), Ether-linked Phosphatidylcholine (PC O-), Phosphatidylethanolamine (PE), Ether-linked Phosphatidylethanolamine (PE O-) Phosphatidylinositol (PI), Sphingomyelin (SM), Triacylglycerol (TAG).

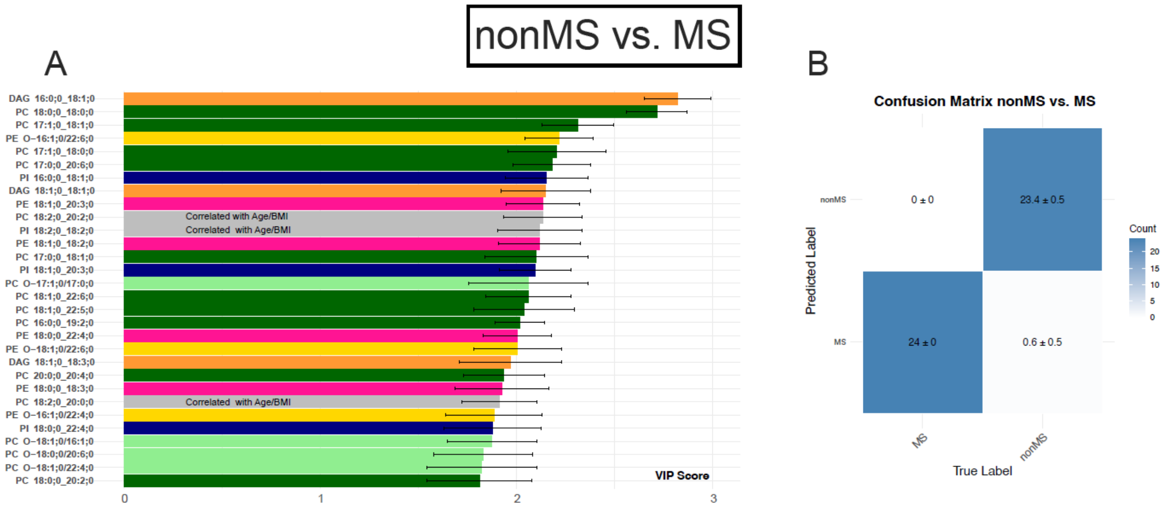

Supplement Figure S7 nonMS vs. MS Comparison

(A) *Bar chart displaying the lipids with the top 30 VIP (Variable influence on projection) of oPLS-DA with SD (nonMS (healthy & OND) vs MS (RRMS & CPMS)).* ***(B)*** *Confusion matrix of the oPLS-DA of nonMS vs MS on the 40% hold out test data set (testing data) ensuring the predictive capability of the important lipids provided by oPLS-DA.*

Cholesteryl ester (CE), Ceramide (Cer), Cholesterol (Chol), Diacylglycerol (DAG), Hexosylceramide (HexCer), Lysophosphatidylcholine (LPC), Ether-linked Lysophosphatidylcholine (LPC O-), Lysophosphatidylethanolamine (LPE), Ether-linked Lysophosphatidylethanolamine (LPE O-), Phosphatidylcholine (PC), Ether-linked Phosphatidylcholine (PC O-), Phosphatidylethanolamine (PE), Ether-linked Phosphatidylethanolamine (PE O-) Phosphatidylinositol (PI), Sphingomyelin (SM), Triacylglycerol (TAG).

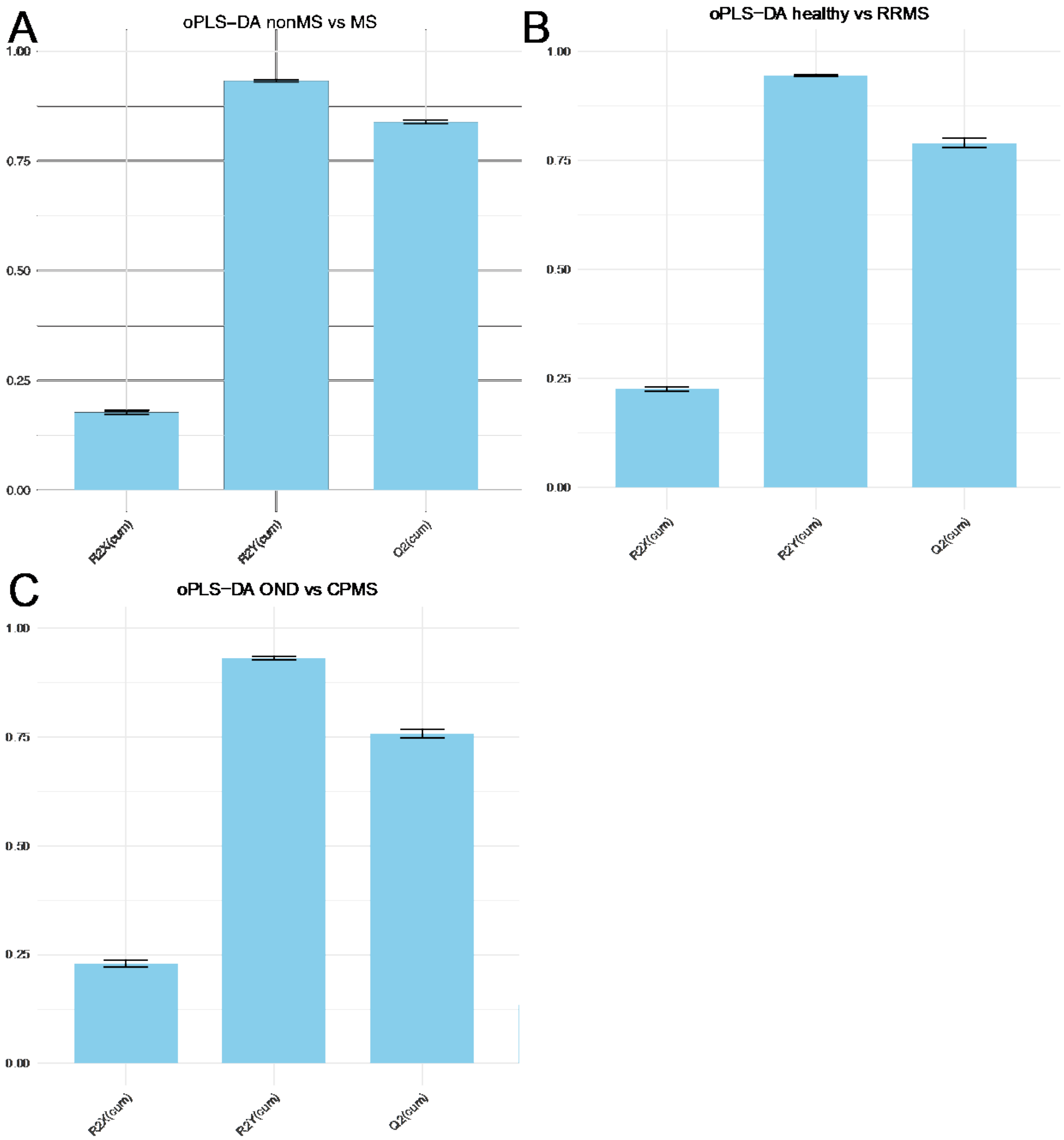

Supplement Figure S8 oPLS-DA Metrics

(A) *oPLS-DA Metrics of nonMS vs MS;* ***(B)*** *oPLS-DA Metrics of healthy vs RRMS ;* ***(C)*** *oPLS-DA Metrics of OND vs CPMS. oPLS-DAs were verified by evaluation of R2X(cum) representing the explained variation in the matrix of predictor variables and R2Y(Cum) which represents the explained variation in the response matrix. The prediction ability of the model is described by Q2 and is calculated using 5-fold cross-validation (Q2(cum)). A sufficient predictive power is assumed at a Q2(cum) value of >0.5. The SD shown was calculated by 20 iterations.*

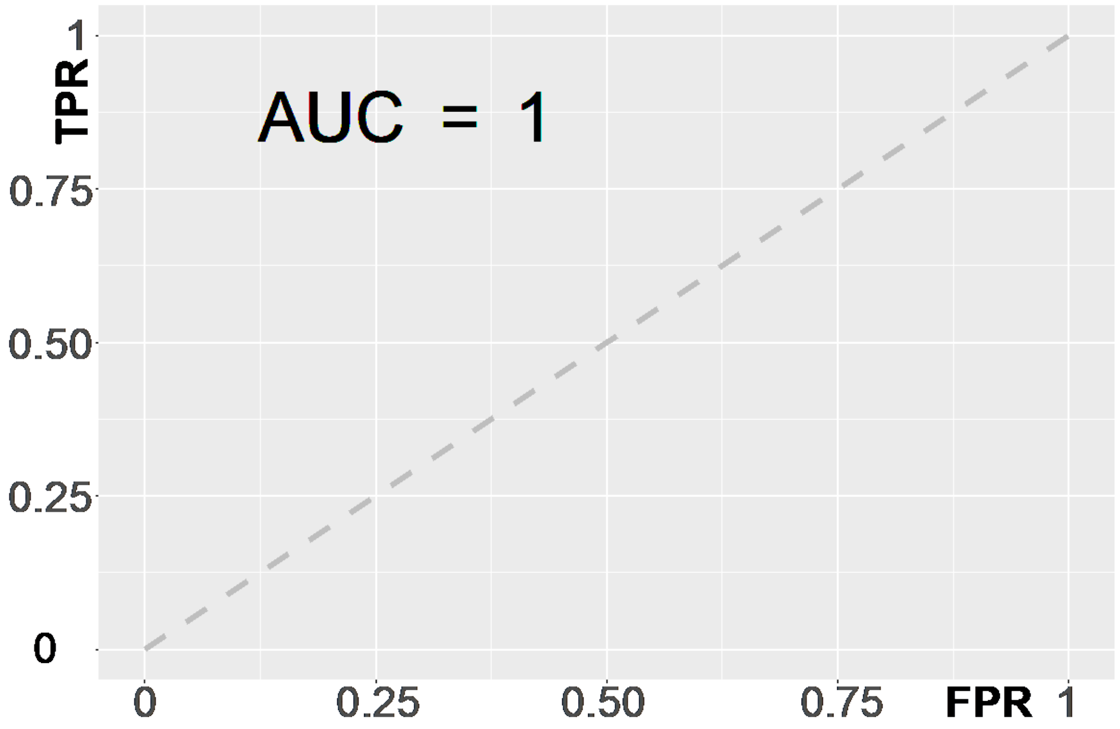

Supplement Figure S9 Receiver Operating Characteristic Curve (ROC-Curve) of the Random Forest

The predictive capability of the Random Forest was evaluated on the hold-out dataset (40%) and demonstrated a prediction with an AUC of 1. This is proving that the lipids highlighted by the Random Forest describe the lipid pattern of MS.

AUC = Area Under Curve; ROC =Receiver Operating Characteristic

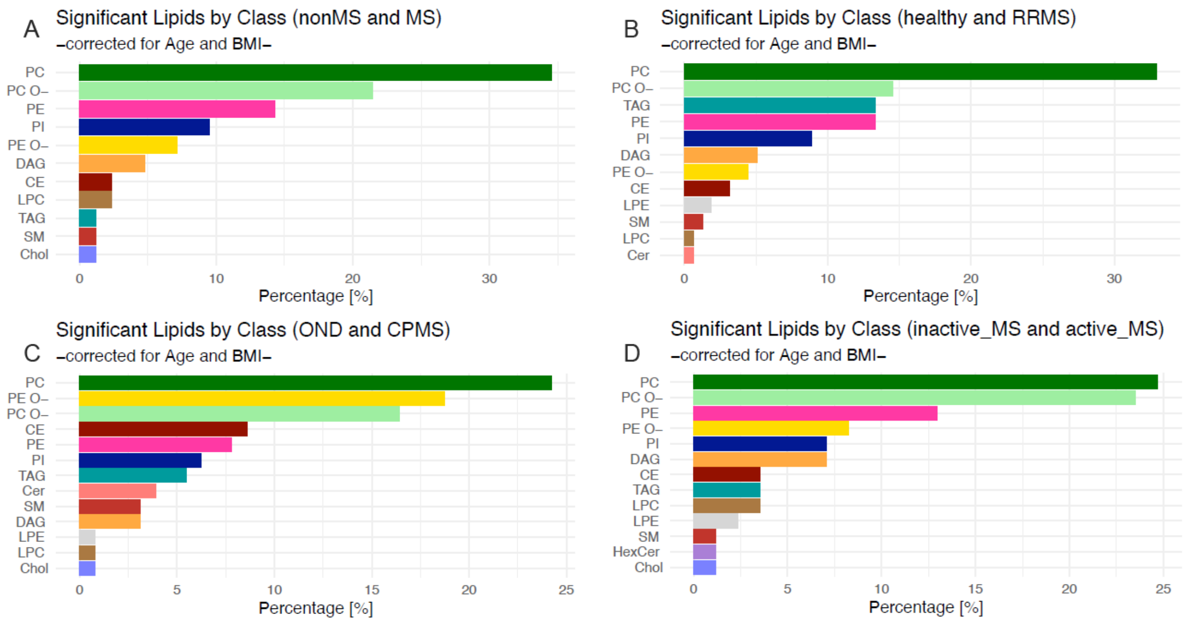

Supplement Figure S10 Percentage of Significant Altered Lipids by Class

These bar plots are visualizing the percentage of significantly altered lipids by lipid class. ***(A)*** *shows comparison of nonMS vs MS;* ***(B)*** *shows healthy vs RRMS;* ***(C)*** *shows OND vs CPMS; and* ***(D)*** *inactive vs active MS.*

The majority of significant changes were observed in the PC class. Interestingly, the comparison of OND vs CPMS against healthy vs RRMS shows that the number of significant changes in PE O- and PC O- differ depending on the compared cohorts.

Cholesteryl ester (CE), Ceramide (Cer), Cholesterol (Chol), Diacylglycerol (DAG), Hexosylceramide (HexCer), Lysophosphatidylcholine (LPC), Ether-linked Lysophosphatidylcholine (LPC O-), Lysophosphatidylethanolamine (LPE), Ether-linked Lysophosphatidylethanolamine (LPE O-), Phosphatidylcholine (PC), Ether-linked Phosphatidylcholine (PC O-), Phosphatidylethanolamine (PE), Ether-linked Phosphatidylethanolamine (PE O-) Phosphatidylinositol (PI), Sphingomyelin (SM), Triacylglycerol (TAG).

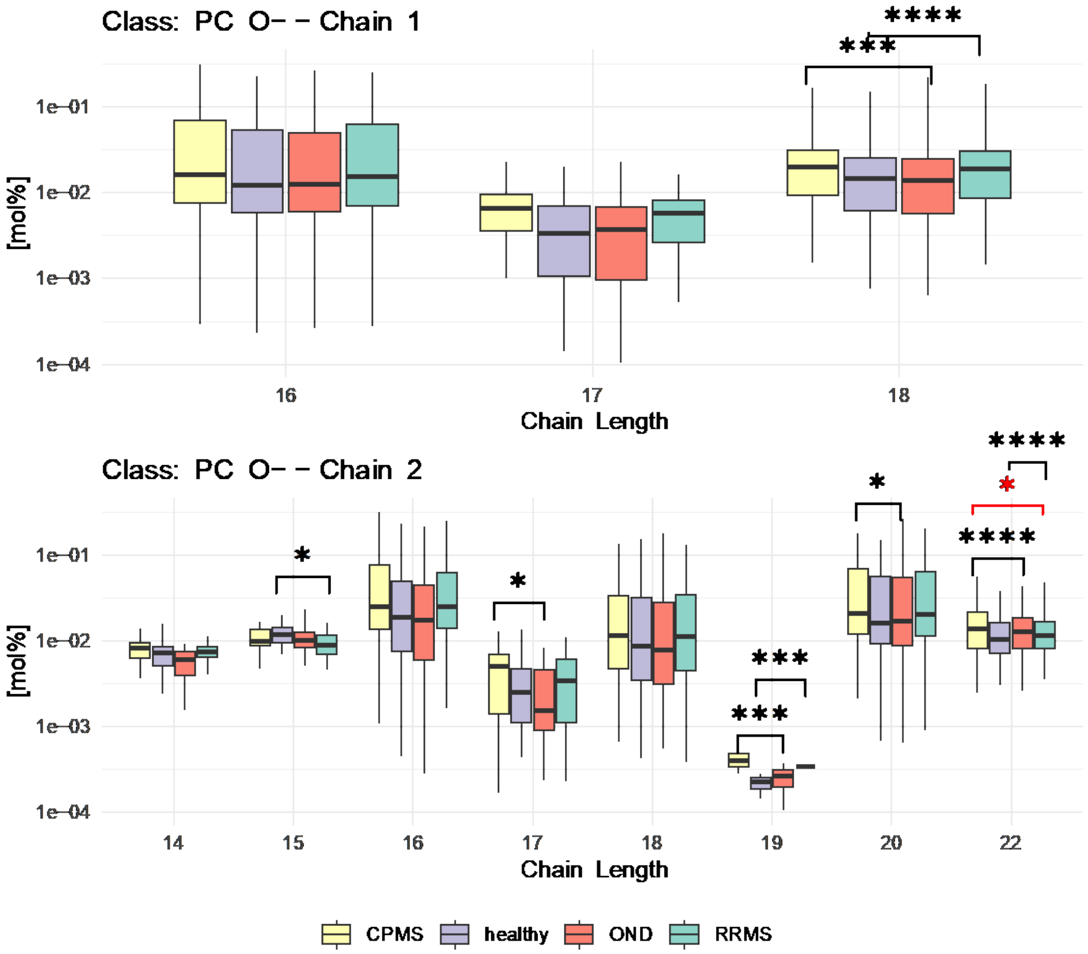

Supplement Figure S11 Chain-Length Analysis Plot

*Bar charts showing the concentration of each acyl chain in relation to the respective lipid class for ether-linked Phosphatidylcholine (PC O-). Statistical significance was determined in Supplement Table 5; * = p-value < 0.05; *** = p-value < 0.001; **** = p-value < 0.0001.*
